# Operando X‐Ray Computed Tomography Reveals the Role of Interfacial Nucleation Nanolayers in Suppressing Mechanical Failure in Zero‐Excess Lithium All‐Solid‐State Batteries

**DOI:** 10.1002/smll.202512284

**Published:** 2026-01-08

**Authors:** Linfeng Xu, James Le Houx, Vyacheslav Kachkanov, Jinsong Zhang, Robin Norbert Wullich, Matthias Fankhauser, Kaspar Löffel, Thomas J. Schmidt, Mario El Kazzi

**Affiliations:** ^1^ PSI Center for Energy and Environmental Sciences Paul Scherrer Institute Villigen Switzerland; ^2^ ISIS Neutron and Muon Source Rutherford Appleton Laboratory Didcot UK; ^3^ The Faraday Institution Harwell Science and Innovation Campus Didcot UK; ^4^ Diamond Light Source Rutherford Appleton Laboratory Didcot UK; ^5^ University of Applied Sciences Northwestern Switzerland (FHNW) Windisch Switzerland; ^6^ Institute for Molecular Physical Science ETH Zurich Zurich Switzerland; ^7^ University of Greenwich Old Royal Naval College, Park Row London UK

**Keywords:** alloy interlayer, all‐solid‐state battery, image subtraction method, Li metal, operando XCT, zero‐excess lithium anode

## Abstract

Lithium metal (LM) and zero‐excess lithium (ZE) anodes offer pathways to increase the energy density of all‐solid‐state batteries (ASSBs). We employ operando X‐ray computed tomography combined with an image subtraction method to visualize lithium plating/stripping morphology, stack mechanical failure, and quantify the lithium reversibility in asymmetric Li_6_PS_5_Cl (LPSC)‐based ASSBs. Lithium metal counter electrode (CE) and copper (Cu) working electrode (WE) emulate LM and ZE interface configurations, respectively. We compare bare Cu and silver‐coated Cu (Ag/Cu) WEs under varying current densities. At 0.25 mA cm^−2^_(WE)_, bare Cu shows edge‐localized and non‐uniform lithium deposition, while Ag/Cu facilitates more uniform lithium spreading, but results in higher first‐cycle irreversibility and lower Coulombic efficiency. Above 0.5 mA cm^−2^_(WE)_, failure in Li|LPSC|Cu cells initiate at the LPSC|Cu interface via spallation cracks. In contrast, Ag preserves interface integrity at the WE despite lithium initially plates at discrete nucleation spots. However, failure shifts to the Li|LPSC interface, where non‐uniform lithium depletion at the CE exposes the underlying Cu, leading to spallation cracks upon subsequent plating. Mechanical finite element simulations support these observations and underscore the critical role of the nucleation layers in mitigating mechanical failure. This study highlights interface engineering as a key strategy to address electro‐chemo‐mechanical degradation in LM‐ and ZE‐ASSBs.

## Graphical abstract

This study employs operando X‐ray computed tomography combined with an image subtraction method to investigate mechanical failure mechanisms in all‐solid‐state batteries with lithium and zero‐excess lithium anodes. By comparing bare and silver‐coated copper current collectors, the results reveal how nucleation nanolayers influence lithium deposition morphology, reversibility, solid electrolyte mechanical integrity, and short‐circuit behavior under varying current densities. These findings emphasize the critical role of interface engineering in mitigating electro‐chemo‐mechanical degradation in zero‐excess lithium all‐solid‐state batteries.

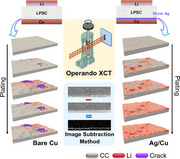

## 1 Introduction

All‐solid‐state batteries (ASSBs), which employ highly ionically conductive, nonvolatile and nonflammable solid electrolytes (SEs), promise improved safety and increased energy density compared to conventional Li‐ion batteries (LiB) using carbonate‐based liquid electrolyte [1, 2, 3]. By enabling the use of thin lithium metal anodes and high‐voltage cathode materials, lithium metal ASSBs (LM‐ASSBs) could theoretically achieve energy density beyond 500 Wh kg^−1^ without compromising safety [4, 5, 6, 7]. Among emerging architectures, zero‐excess lithium all‐solid‐state batteries (ZE‐ASSBs) are particularly attractive. In this design, the cathode supplies lithium, which plates onto the current collector (CC) during the first charge and is stripped during discharge. ZE‐ASSBs are promising for maximizing energy density and simplifying manufacturing [8, 9, 10, 11, 12, 13]. Nevertheless, the practical realization of both thin LM‐ or ZE‐ASSBs remains challenging. Key limitations include unpredictable lithium morphology evolution, large volume changes compromising stack and current collector mechanical stability, and void formation that impedes lithium diffusion and causes contact loss between the SE and Li, leading to dendrite growth, short circuits, and failure. In addition, the interfacial (electro‐)chemical reactivity with the SE [14, 15] and the formation of isolated lithium result in the formation of electrochemically inactive lithium and insufficient Coulombic efficiency (CE) during cycling, pose critical challenges to the long‐term stability of ZE‐ASSBs [16, 17, 18].

Substantial efforts have been devoted to acquiring a comprehensive understanding of the lithium plating and stripping behavior, and lithium dendrite formation mechanisms as one of the primary failure modes in ASSBs. In LM‐ASSBs, dendrite formation during plating has been linked to factors such as surface flaws [19] microstructure [20] grain boundaries [21, 22], and the electronic conductivity of the SEs [23]. During lithium stripping at low pressure, voids can be formed at the Li/SE interface, which reduces the interfacial contact area and creates large inhomogeneous current densities that accelerate lithium dendrite growth and penetration into the SEs [24, 25, 26, 27]. Mechanistic studies specifically on ZE‐ASSBs have revealed additional complexity. For instance, Lee et al. [28] have demonstrated different mechanisms restricting the stripping of in situ plated lithium, depending on lithium thickness and current densities. While Lewis et al. [29] highlighted the detrimental impact of isolated lithium regions on the CC caused by local lithium depletion toward the end of stripping on the electrochemical performance.

To address these challenges, surface engineering of the current collector has been extensively investigated to reduce the lithium nucleation barrier and improve the wettability [30, 31]. Strategies includes for instance, (i) the introduction of Ag or Ag‐C composite particles interlayers with micron‐scale thickness [32, 33], or (ii) deposition of thin nucleation layers (100s of nm) of lithiophilic and lithium‐soluble materials (e.g., Ag, Zn, Au, Mg, etc.) [34], metal fluoride [35], or bilayers with both lithophilic soluble (Mg) and lithiophobic insoluble (W) materials [36], with the purpose to improve the lithium diffusion, mechanical interface stability at low pressure, prevent lithium reaction with the SE and block the lithium dendrite growth. It is also worth mentioning that thick intermetallic alloy anodes such as Ag‐Li [8] or Li‐Mg [37] have been found beneficial to improve the electrochemical performance also in LM‐ASSBs. In particular, Li‐Mg has been proven to mitigate uneven lithium deposition, maintain a stable interface, and effectively prevent macroscopic pore formation at low pressure. This, in turn, helps suppress contact loss and unwanted local current constriction [37].

These advances, however, have largely focused on LM‐ASSBs. For ZE‐ASSBs, a mechanistic understanding that directly links lithium morphology and solid electrolyte mechanical stability to the ultimate mode of cell failure, particularly when comparing bare and surface‐modified current collectors, remains elusive. In particular, the process visualization in real‐time on realistic systems. The lithium plating‐stripping evolution occurs at the deeply buried interfaces, which is highly challenging to probe and visualize using conventional post‐mortem analytical methods.

Several characterization techniques have been employed to study the lithium metal dynamics in ASSBs, such as ex situ focused‐ion beam scanning electron microscopy (FIB‐SEM) [38, 39], operando optical microscopy [40] and operando neutron imaging [41]. However, each comes with a trade‐off. For example, (i) ex situ FIB‐SEM, despite its capability of providing high‐resolution images, it is destructive, probes localized area and lacks insights into the dynamic evolution of lithium morphology, (ii) optical microscopy is often limited to model systems, unsuitable for probing buried interfaces and limited on system‐wide insights, and (iii) operando neutron imaging, although being highly sensitive to lithium metal, often suffers from poor spatial resolution.

In comparison, operando X‐ray computed tomography (XCT) acquired with a high‐brightness synchrotron X‐ray source is non‐destructive and capable of visualizing the entire ASSB stack with high spatial and temporal resolution. In recent years, operando XCT has gained popularity in monitoring lithium metal behaviors and visualizing relevant mechanical failure mechanisms in ASSBs. For instance, Ning et al. [42, 43] have visualized the lithium dendrite initiation and propagation into the SE and demonstrated that the lithium dendrite growth drives the crack ahead. Void formation during lithium stripping has also been imaged through operando XCT measurements [25]. Furthermore, recently operando XCT measurements have been applied to monitor the lithium morphology changes in ZE‐ASSBs [44].

In this work, we conducted operando synchrotron XCT measurements with sufficient spatial resolution and phase contrast to elucidate, in real time, the mechanical failure mechanisms associated with lithium plating and stripping in asymmetric Li_6_PS_5_Cl (LPSC)‐based ASSBs. The cells are composed of 50‐µm‐thick lithium coated on copper as the counter electrode (CE) and copper foil as the working electrode (WE) to mimic both lithium metal and zero‐excess lithium interface configurations, respectively. Two cell architectures were studied: Li|LPSC|Cu using bare copper foil (denoted Cu) and Li|LPSC|Ag/Cu featuring 50 nm silver‐coated copper foil (denoted Ag/Cu) as current collectors. These cells were cycled under both low (0.25 mA cm^−2^_(WE)_) and high (≥0.5 mA cm^−2^_(WE)_) current densities. The operando XCT aimed to shed light on (i) the morphological evolution of lithium plating and stripping, (ii) the mechanical stability of the LPSC, (iii) quantification of lithium reversibility, and (iv) the cell short‐circuits initiating from different interfaces, including Li|LPSC and LPSC|CC.

Furthermore, we developed an image processing workflow based on the image subtraction method (ISM) to improve the accuracy of quantitative analysis of lithium plating and stripping near the solid SE|CC interfaces. Accurate visualization and quantification of lithium metal in this configuration using XCT is highly challenging due to several factors: the presence of dense surrounding metals (e.g., Cu, Ag, and stainless‐steel dies from the cell assembly), low‐attenuation phases such as voids or porous regions in the SE, and partially lithium‐filled voxels with grayscale values (GSVs) that significantly deviate from those of pure lithium metal. ISM allows for enhanced detection and analysis of lithium morphology and distribution in these complex environments.

Finally, based on the distinct lithium plating morphologies observed on bare Cu and Ag‐coated Cu, we performed mechanical finite element simulations to reproduce the growth of lithium islands with two representative geometries at the SE|Cu and the SE|Ag/Cu interfaces. These simulations provide insight into the interfacial stress evolution during lithium deposition and implications for mechanical instability in LPSC SE.

## 2 Results and Discussion

### 2.1 Experimental Set‐Up and Electrochemical Performance

Building on our previous work [45] we employed a custom‐designed operando tomography cell dedicated to ASSBs measurements (Figure S1a), which was previously validated using SnO_2_ anode materials. The entire external frame is made of low X‐ray absorbing material poly(methyl methacrylate) (PMMA). Three pillars (Φ = 3 mm) evenly distributed around the cell body were sufficient to withstand the applied pressure and minimize beam absorption, as only one pillar would attenuate the beam at a time while the cell rotates through 180° during the measurement. As a result, we did not observe any traceable artifacts potentially from the pillar design in the tomography scan (Figure S1b). The cell was mounted on a multi‐directional adjustable sample stage and carefully aligned with the synchrotron X‐ray beam and the detector. It was connected to a potentiostat, enabling simultaneous electrochemical cycling and XCT measurements (Figure S1c).

The ASSB stack was assembled inside a polyether ether ketone (PEEK) tube with inner diameter of 3 mm and comprised three layers: a 50 µm lithium film coated on copper foil serving as the counter electrode (CE), a dense pellet of Li_6_PS_5_Cl (LPSC) as the SE separator, and a copper current collector (CC), with and without a silver coating, as the working electrode (WE). A stack pressure of approximately 20 MPa was maintained throughout the experiments by a 3 mm stainless steel die.

Two cells with this architecture were investigated (Figure S2), differing only in the current collector of the WE. The first cell used bare copper foil (denoted as Li|LPSC|Cu), while the second cell used copper foil coated with a 50 nm silver nucleation layer (denoted as Li|LPSC|Ag/Cu). The silver was deposited via DC‐magnetron sputtering, with the thickness confirmed by cross‐sectional SEM (Figure S3). Plan view SEM measurements revealed that the silver coating minimally affects the microscale surface morphology compared to the bare Cu (Figure S4a), although high magnification images confirmed silver grain sizes of 10–50 nm (Figure S4b).

**FIGURE 1:**
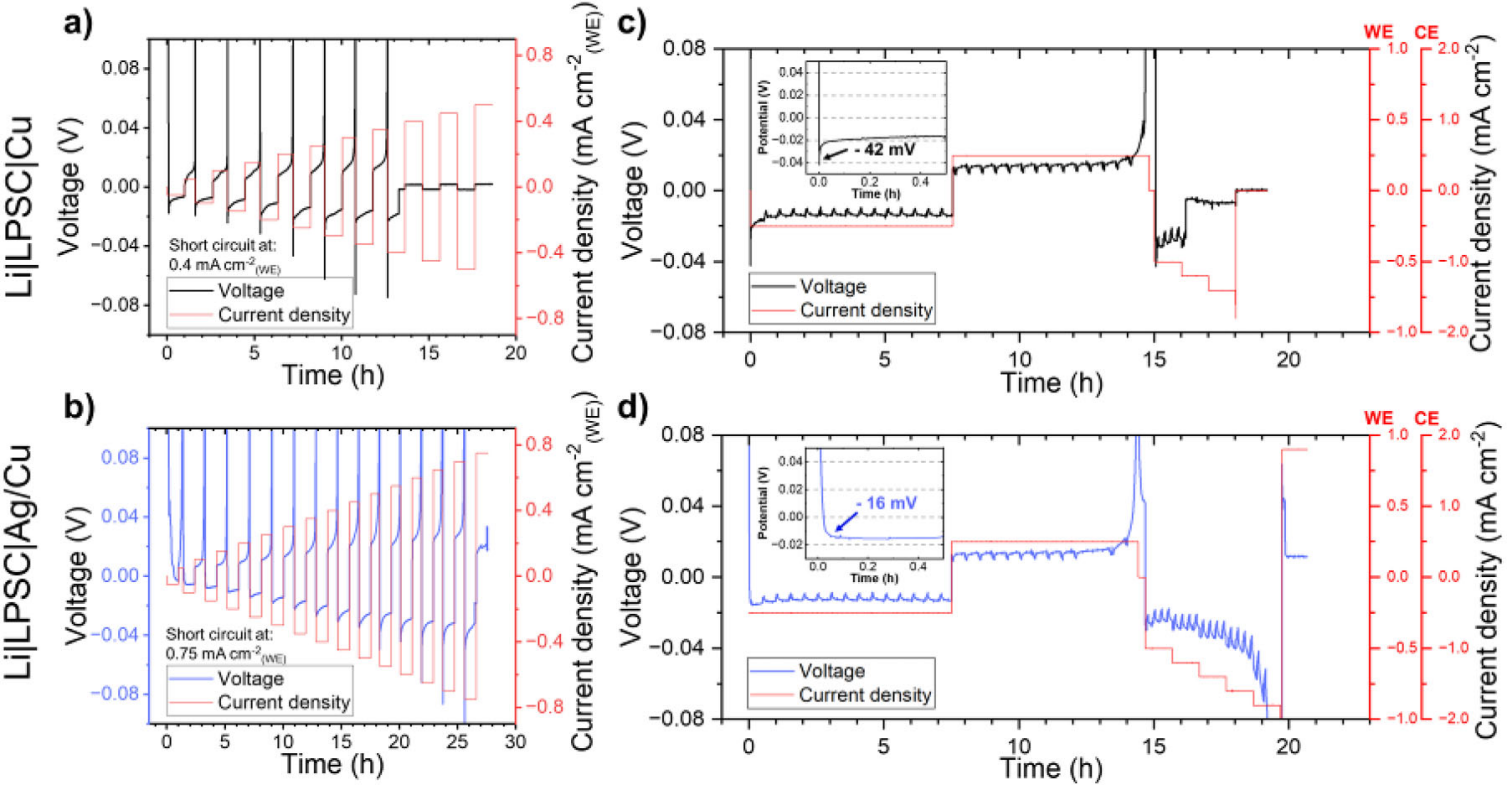
Critical capacity and current density test of the (a) Li|LPSC|Cu and (b) Li|LPSC|Ag/Cu cells. Voltage profiles of galvanostatic plating and stripping during operando XCT measurements performed on the (c) Li|LPSC|Cu and (d) Li|LPSC|Ag/Cu cells.

Prior to operando XCT measurements, a critical capacity and current density (CCCD) test was conducted on both Li|LPSC|Cu and Li|LPSC|Ag/Cu cells to evaluate their electrochemical performance. Increasing current densities and areal capacities were applied in successive steps with a half‐cycle time of 1 h until short circuits occurred. The Li|LPSC|Cu cell shorted during plating at 0.4 mA cm^−2^_(WE)_ (Figure 1a), whereas the Li|LPSC|Ag/Cu cell exhibited significantly improved stability, shorting at 0.75 mA cm^−2^_(WE)_ (Figure 1b). For clarity, throughout this work, unless otherwise specified, the terminologies “plating” and “stripping” as well as the current density and areal capacity values, refer to those on the WE (the zero‐excess lithium current collector). The current density applied on the CE is approximately twice of the one applied on the WE, as the area of lithium at the CE is approximately 50% of the area of Cu on the WE. It is worth noting that during the first plating, the Li|LPSC|Cu cell showed a sharp voltage drop from 1.7 to −18 mV, while the Li|LPSC|Ag/Cu cell, exhibited distinct plateaus near 0.6 V, 50 mV, 35 mV, and 7 mV, versus Li^+^/Li with the voltage dropping from 1.8 to −3 mV, which are characteristic of Ag‐Li alloying redox processes (indicated by blue arrows in Figure S5). The alloying signatures are progressively diminishing in subsequent cycles, indicating that the lithium‐rich surface serves as the host for further plating and stripping (Figure S5).

The electrochemical cycling protocol during the operando XCT measurements was designed based on the CCCD test results using a two‐stage approach. First, to establish a baseline, an initial cycle was conducted at a low current density of 0.25 mA cm^−2^_(WE)_ (at the working electrode), safely below the critical short‐circuiting point for either cell. This was applied for a half‐cycle time of 7.5 h (areal capacity of 1.875 mAh cm^−2^_(WE)_) corresponding to a lithium equivalent plating and stripping thickness of 8.1 µm _(WE)_. Second, to deliberately probe the failure mechanisms, higher current densities were applied for the second plating cycle, starting at 0.5 mA cm^−2^_(WE)_ and increasing by 0.1 mA cm^−2^_(WE)_ every hour, until the intentionally induced short circuit occurred.

Figure 1c,d shows the voltage profiles and the current densities recorded on both cells during the operando XCT measurements. The measurements exhibit periodic voltage spikes caused by the periodic XCT scan acquisition. These artifacts are not observed under standard laboratory cycling conditions (Figure S6). At the start of the first plating, the Li|LPSC|Cu cell showed a higher nucleation overpotential (≈42 mV) than the Li|LPSC|Ag/Cu cell (≈16 mV). The reduced nucleation overpotential on the Ag‐coated surface arises from the Ag‐Li alloy redox reactions, known to facilitate Li metal nucleation [10, 31].

During the first plating and stripping cycle, neither of the cells experiences a short circuit, and the potentials remained stable until the end of stripping, where a sharp potential rise was observed, indicating a lack of electrochemically active lithium at the WE. In the second plating, the Li|LPSC|Cu cell was shorted within 10 min after increasing the current density to 0.6 mA cm^−2^_(WE)_ and plating a total areal capacity of 0.6 mAh cm^−2^_(WE)_. Conversely, the Li|LPSC|Ag/Cu cell sustained plating up 0.9 mA cm^−2^_(WE)_ without shorting, although the potential sharply increased due to the non‐uniform lithium stripping at the CE. Notably, the Li|LPSC|Ag/Cu cell short‐circuited 7 min after reversing the current direction to stripping lithium from the WE at 0.9 mA cm^−2^_(WE)_ with an areal capacity of merely 0.1 mAh cm^−2^_(WE)_. It is important to mention that throughout the entire operando measurements, the XCT scans were acquired periodically without interrupting the electrochemical cycling.

### 2.2 Insights from Operando XCT Measurements

The ASSB stack structure is illustrated by a representative cross‐sectional XCT image slice (Figure S7), in which distinct phases of interest are identified by their grayscale values (GSVs). Lithium appears darkest, the sulfide SE separator displays intermediate grey, and the copper current collector along with the stainless‐steel dies located on both sides of the stack appear brightest, consistent with increasing material densities and X‐ray attenuation coefficients.

**FIGURE 2:**
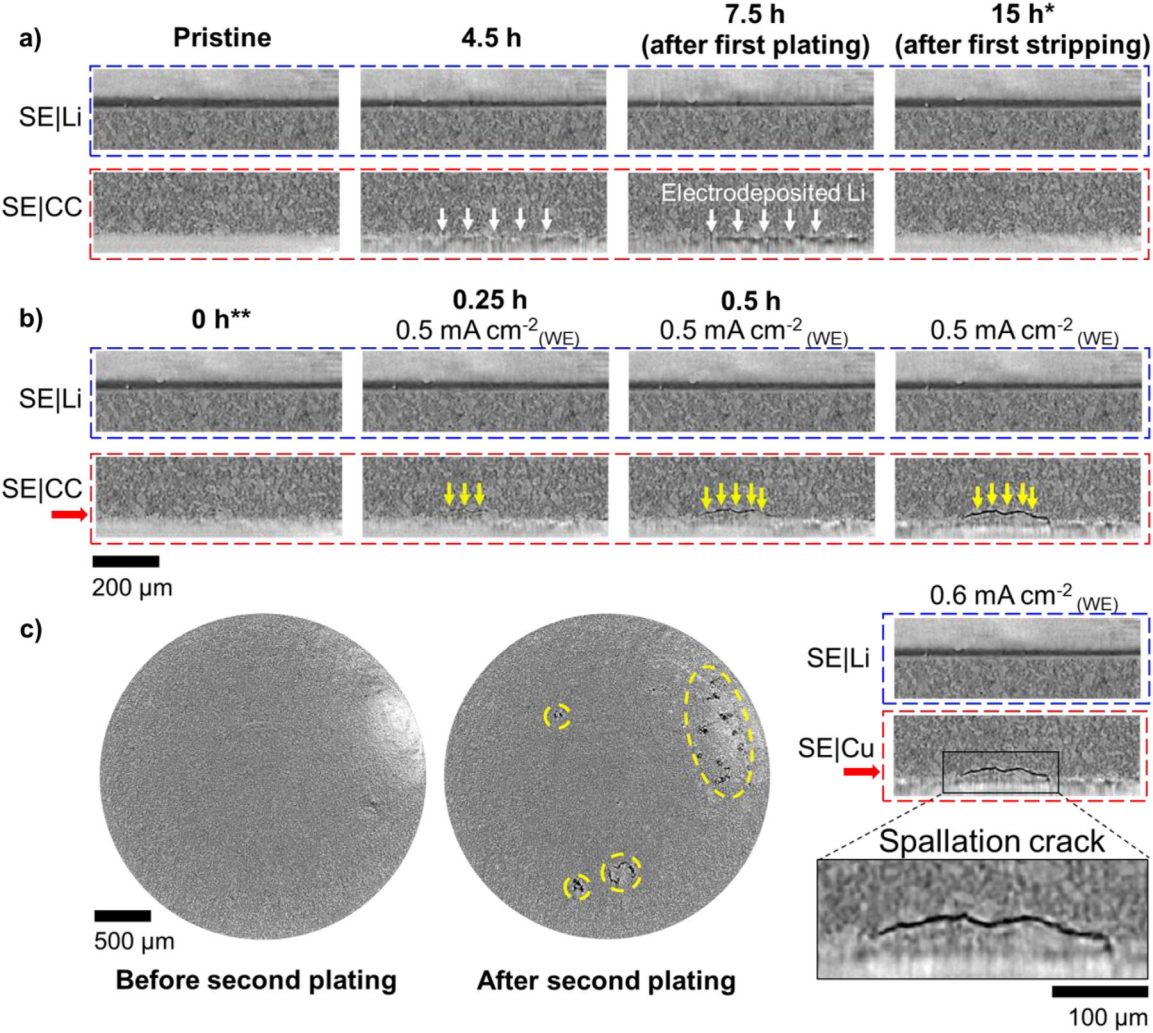
Cross‐sectional XCT image slices of a fixed region near the SE|Li and SE|CC interfaces in the Li|LPSC|Cu cell (a) during first plating and stripping at low current density of 0.25 mA cm^−2^_(WE)_ (from left to right: pristine, 4.5, 7.5, and 15 h after the start of plating), and (b) during second plating at higher current densities (from left to right: 0, 0.25, 0.5, and 1.25 h after the start of plating). The black outlined rectangle shows a magnified view of the spallation crack on the Cu electrode after the second stripping. (c) In‐plane image slices taken parallel to and 5 pixels (≈8 µm) above the SE|CC interface in the Li|LPSC|Cu cell before (left) and after (right) the second plating, with the slice positions indicated by red arrows in (b), and the yellow dashed ovals suggesting the positions of spallation cracks. *In the first cycle, we note 15 h as the end of the plating for convenience, even though both cells stopped stripping slightly before 15 h due to irreversibility. **All the time coordinates here used for studying the second plating and stripping stand for the time passed after the start of the second plating.

**FIGURE 3:**
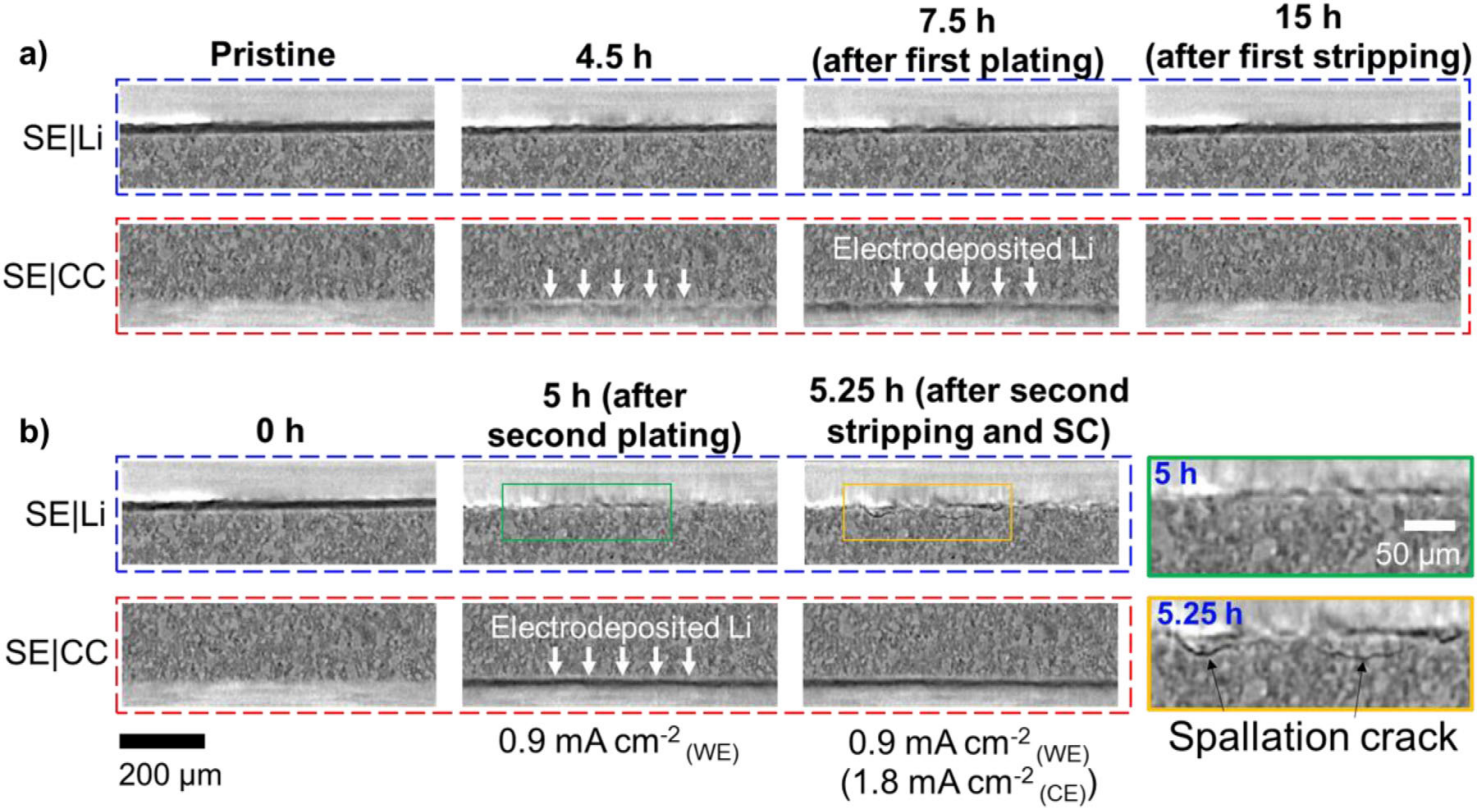
Cross‐sectional XCT image slices of a fixed region near the SE|Li and SE|CC interfaces in the Li|LPSC|Ag/Cu cell (a) during first plating and stripping at low current density of 0.25 mA cm^−2^_(WE)_ (from left to right: pristine, 4.5, 7.5, and 15 h after the start of plating), and (b) during second plating and stripping at higher current densities (from left to right: 0, 5, and 5.25 h after the start of plating). The black outlined rectangle shows a magnified view of the spallation crack on the Li electrode after the 2nd stripping.

The structural and morphology evolution near the SE|Li (blue dashed boxes) and SE|CC (red dashed boxes) interfaces during the first plating and stripping cycle at low current density of 0.25 mA cm^−2^_(WE)_ is displayed for both Li|LPSC|Cu (Figure 2a) and Li|LPSC|Ag/Cu (Figure 3a) cells. As expected, under these mild conditions, the lithium thickness on the CE visibly decreased by approximately 14 µm during the first plating on the WE and largely recovered after the first stripping on both cells. In the case of a bare copper current collector, a wrinkle morphology of the plated lithium (indicated by white arrows) (Figure 2a) was evident at plating completion. This is attributed to lateral heterogeneity in lithium distribution, where the lithium does not fully cover the surface of the bare copper. Early‐stage wrinkling was also visible at 4.5 h, although contrast was weak due to the high attenuation coefficient of the nearby stainless‐steel and copper current collectors. Due to the high and comparable densities of the Cu foil (8.96 g cm^−3^) and the stainless‐steel plunger (8.0 g cm^−3^), the 20‐µm‐thick Cu foil is not distinguishable in the XCT images. Consequently, it is difficult to determine whether the Cu was mechanically deformed or if lithium was plated into or beneath the Cu foil. We believe that the observed wrinkle‐like morphology results from a synergistic effect of SE surface roughness and inhomogeneous lithium plating on Cu. Moreover, under a cycling pressure of 20 MPa, significant mechanical deformation of the Cu foil is unlikely. In contrast, the plated lithium on Ag‐coated current collector (Figure 3a), appeared more uniform, fully covering the surface of the current collector, at both early and late plating stages. The 50 nm Ag layer could also not be visualized under the current experimental setup. However, previous studies have provided some relevant insights into the lithium deposition mechanisms involving Ag interlayers. For instance, Sandoval et al. [10] employed a 100 nm Ag interlayer with LPSC and observed Ag‐rich particles nonuniformly distributed throughout the plated lithium layer. Similarly, Ko et al. [46] used a 200 nm Ag interlayer with garnet SE and reported that the plated lithium is passing through the Li‐Ag alloy layer, while for other metal interlayers with Cu, Au, and Zn the lithium plating occurs at the SE|interlayer interfaces. Sohn et al. [47] developed an Ag‐ZnO dual‐seed interlayer with LPSC and found spatially uniform incorporation of Ag throughout the plated lithium layer after the first deposition.

Subtle morphological differences between pristine and post‐stripping images made it challenging to identify residual lithium or structural changes on the WE in both cells. Nonetheless, it is evident that cycling at low current density preserve the mechanical integrity of the stack; no obvious cracks formation was observed in the SE separator, and the lithium plating and stripping process remained visually reversible. This is consistent with the galvanostatic cycling results (Figure 1c,d), which showed stable operation without short‐circuiting under these conditions.

Figures 2b and 3b pictured the structural and morphological evolution during the second plating and stripping cycle at high current densities above 0.5 mA cm^−2^_(WE)_, for the Li|LPSC|Cu and the Li|LPSC|Ag/Cu cells, respectively. At harsh plating condition, in the case of the Li|LPSC|Cu cell, during the plating at 0.5 mA cm^−2^_(WE)_, spallation cracks appeared in the SE separator near the LPSC|Cu interface, which starts to be visible after 15 min (indicated by yellow arrows in Figure 2b) corresponding to approximately 0.54 µm of plated lithium, exhibiting conical geometrical shape similar to prior reports in Li|LPSC|Li cells [42]. These spallation cracks manifested at multiple locations (indicated by yellow ovals in Figure 2c). Interestingly, the spallation cracks progressively increase in size, reaching their maximum in our experiment after 60 min. However, despite the crack's formation and the plating of approximately ≈2.16 µm of lithium the cell didn't short at 0.5 mA cm^−2^_(WE)_. Nevertheless, the cell shorted 15 min after increasing the current to 0.6 mA cm^−2^_(WE)_, corresponding to additional lithium plating of approximately 0.65 µm. Figure S8 presents GSV line profiles extracted from 60 to 75 min across the spallation cracks. No clear progressive increase in greyscale values is observed within the cracks during plating as was reported by Ning et al. [42]. This likely indicates that the lithium fills the cracks from the early stages of the plating process.

Nonetheless, no transversal cracks bridging the WE and CE were observed after the cell short‐circuited, possibly due to their sub‐micron scale, which is too challenging to visualize with our imaging experimental set‐up with a voxel size of 1.625 × 1.625 × 1.625 µm3. Achieving micron‐size transversal cracks requires much higher current densities as reported by Ning et al. [42].

In contrast, for the Li|LPSC|Ag/Cu cell, throughout the entire plating on the Ag‐coated copper current collector, no spallation cracks were captured near the LPSC|Ag/Cu interface, consistent with the absence of a short circuit (Figure 1d). A uniform plated lithium layer was maintained despite current densities reaching up to 0.9 mA cm^−2^_(WE)_ and a deposited lithium equivalent thickness of approximately 15 µm. At the end of the second plating, a sharp overpotential increase is observed (Figure 1d), correlating with the XCT images at 5 h (Figure 3b) acquired at the SE|Li interface. These images reveal that approximately 30 µm of lithium was non‐uniformly stripped at such high current density (1.8 mA cm^−2^_(CE)_), creating spots on the copper current collector fully depleted in lithium. On the one hand, we could see that the current collector of the CE has been exposed and is in direct contact with the LPSC. On the other hand, we noticed that there was still inactive lithium remaining on the CE, which indicates that the stripping from the CE was incomplete and sluggish at 1.8 mA cm^−2^_(CE)_. Subsequent plating at 1.8 mA cm^−2^_(CE)_ on the CE induced a rapid short circuit in the Li|LPSC|Ag/Cu cell, again well correlated with the appearance of spallation cracks at the SE|Li interface. It can be visualized in the magnified image in Figure 3b, which has a similar morphology as the spallation cracks in Figure 2b. It confirms that the spallation crack formation mechanism is the same in both cells, induced by the lithium plating directly on bare copper spots, where the lithium is locally plated, inducing localized strain accumulation at the surface of the SE and ultimately leading to its fracture. This result and observation demonstrate that in Li|LPSC|Ag/Cu system, the cell cycling performance limitation (short‐circuiting) comes from the LPSC|Li interface while the LPSC|Ag/Cu interface preserves its mechanical integrity.

### 2.3 Image Processing and Analysis

Although the gray‐scale images illustrated above provide valuable information regarding the morphology and structural evolution within the ASSBs stack, it remains qualitative and somewhat limited to visualize and quantifying the lithium. Quantitative analysis is challenging because lithium's low density and weak X‐ray attenuation make it hard to distinguish from voids or porous SE regions. Dense metals such as Cu and Ag also cause artifacts during reconstruction via beam hardening, scattering, and photon starvation. Finally, partially lithium‐filled voxels exhibit grayscale values (GSVs) that differ significantly from pure lithium metal voxels due to varying average densities, complicating reliable lithium segmentation using a conventional GSV‐based method.

To overcome these challenges, we developed, validated, and applied an image subtraction method (ISM)‐based processing workflow to quantitatively analyze the dynamic lithium plating and stripping mechanisms near the SE|CC interfaces. ISM generates a difference image by subtracting one time‐resolved‐image from another, thereby highlighting systematic morphological changes over time. This approach is particularly advantageous for dynamic electrochemical systems and has been applied in prior studies to visualize evolving phases over time [41, 48]. Before applying the subtraction, images of the same time series were spatially aligned using a stable region within the bulk SE to eliminate any possible sample drift during operando XCT acquisition. To verify the applicability of ISM, we selected an inactive region corresponding to the PEEK tube surrounding the ASSB stack (Figure S9) to assess the scan stability across the entire time series. As shown in Figure S10, the GSVs within the selected region remain remarkably stable during the measurements of both cells, confirming the suitability of the ISM for subsequent analysis. Besides, the GSV variation in the region lies in a reasonable range (significantly lower than the average GSV difference between CC and Li phases), suggesting limited impact of signal noise.

**FIGURE 4:**
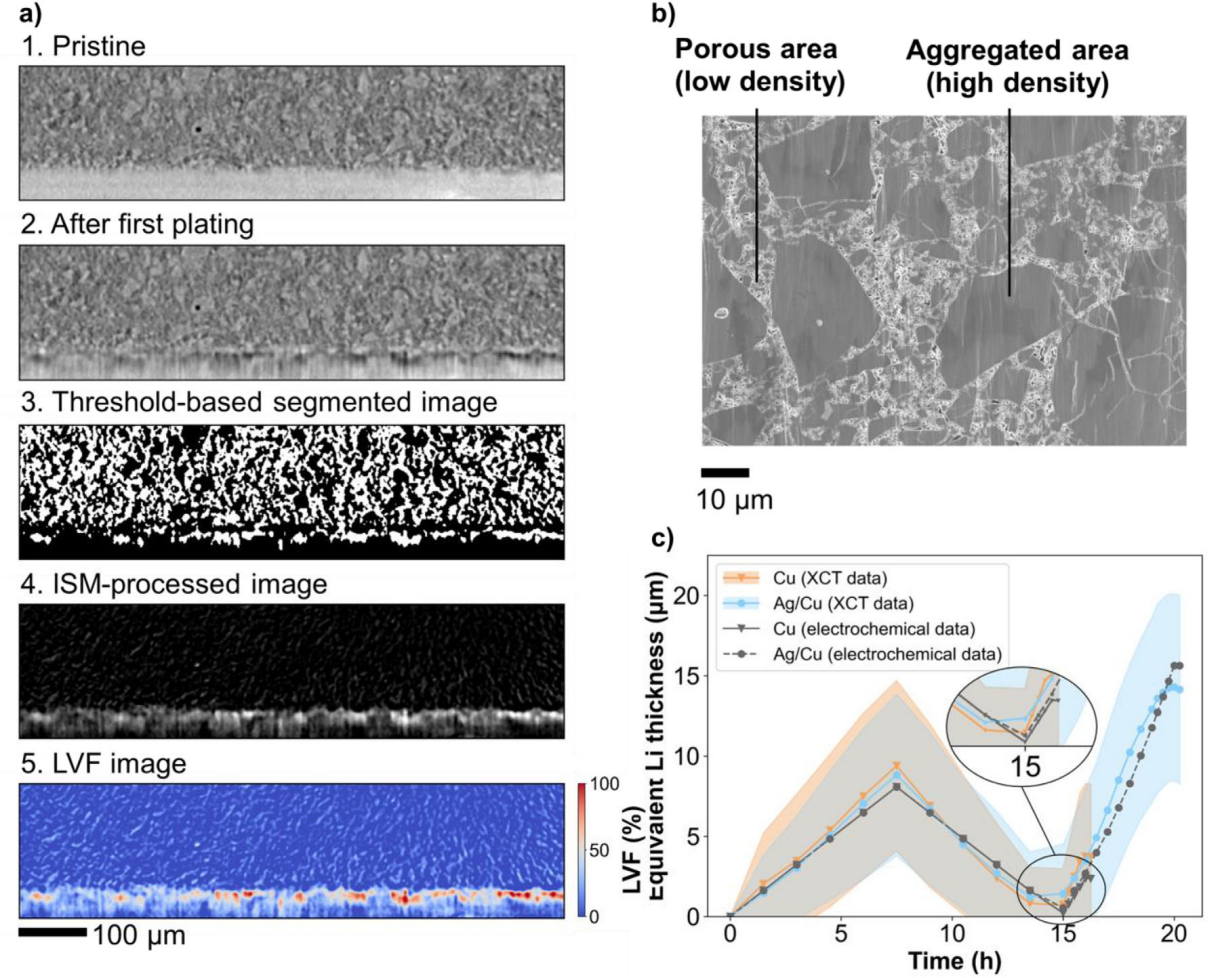
(a) Cross‐sectional XCT image slices of a fixed region near the SE|CC interface in the Li|LPSC|Cu cell (a.1) at pristine state, (a.2) after first plating, (a.3) processed with GSV‐based segmentation, and (a.4) ISM‐processing between (a.1) and (a.2) images. (a.5) LVF image after normalization on (a.4) image. (b) Cross‐sectional SEM image of the LPSC separator, suggesting the existence of porous area with lower density and an aggregated area with higher density. (c) Evolution of the equivalent Li thickness on both cells, including the values calculated from the XCT data and the electrochemical data. The shaded area indicates the range of equivalent thickness, characterized by the mean value and standard deviation of the thickness distribution.

Comparing ISM results with conventional GSV‐based segmentation highlights the advantages of the former in visualizing the plated/stripped lithium. Figure 4a.1,a.2 refer to a fixed region near the SE|CC interface in the Li|LPSC|Cu cell before and after the first plating of 8.1‐µm‐thick lithium, respectively. GSV‐based segmentation applied to the XCT image in Figure 4a.2 results in the image presented in Figure 4a.3, where the lithium phase is marked in white. However, with such an image processing method, a significant fraction of the SE is erroneously classified as the lithium phase. This misclassification is attributed to the heterogeneous internal structure of the LPSC separator, as revealed by the cross‐sectional SEM image of the LPSC separator fabricated under the same conditions (Figure 4b). The separator comprises relatively dense LPSC chunks (10–30 µm particle size), alongside regions of smaller LPSC particles with higher porosity and lower density. These low‐density LPSC regions have confused the segmentation algorithm with the GSVs similar to the lithium phase. Moreover, we also noticed that the plated lithium on the copper current collector is not fully captured by this segmentation method.

In contrast, ISM applied between the after‐plating image (Figure 4a.2) and the pristine image (Figure 4a.1) results in the image reported in Figure 4a.4, which effectively improved the plated lithium layer visualization without interference from the SE phase, consistent with plated lithium being the sole dynamic process with significant morphological change in this region of interest. The ISM image was further normalized by the average GSV difference between the lithium phase and the CC phase. The image after normalization is denoted as lithium volume fraction (LVF) image (Figure 4a.5), where each pixel's value varies from 0 (unchanged CC phase pixels) to 100% (completely plated pixels), corresponding to the degree of transformation from the CC to the lithium phase. In the LVF image, the primary lithium‐plated region appears in red, while unchanged regions are colored in blue.

**FIGURE 5:**
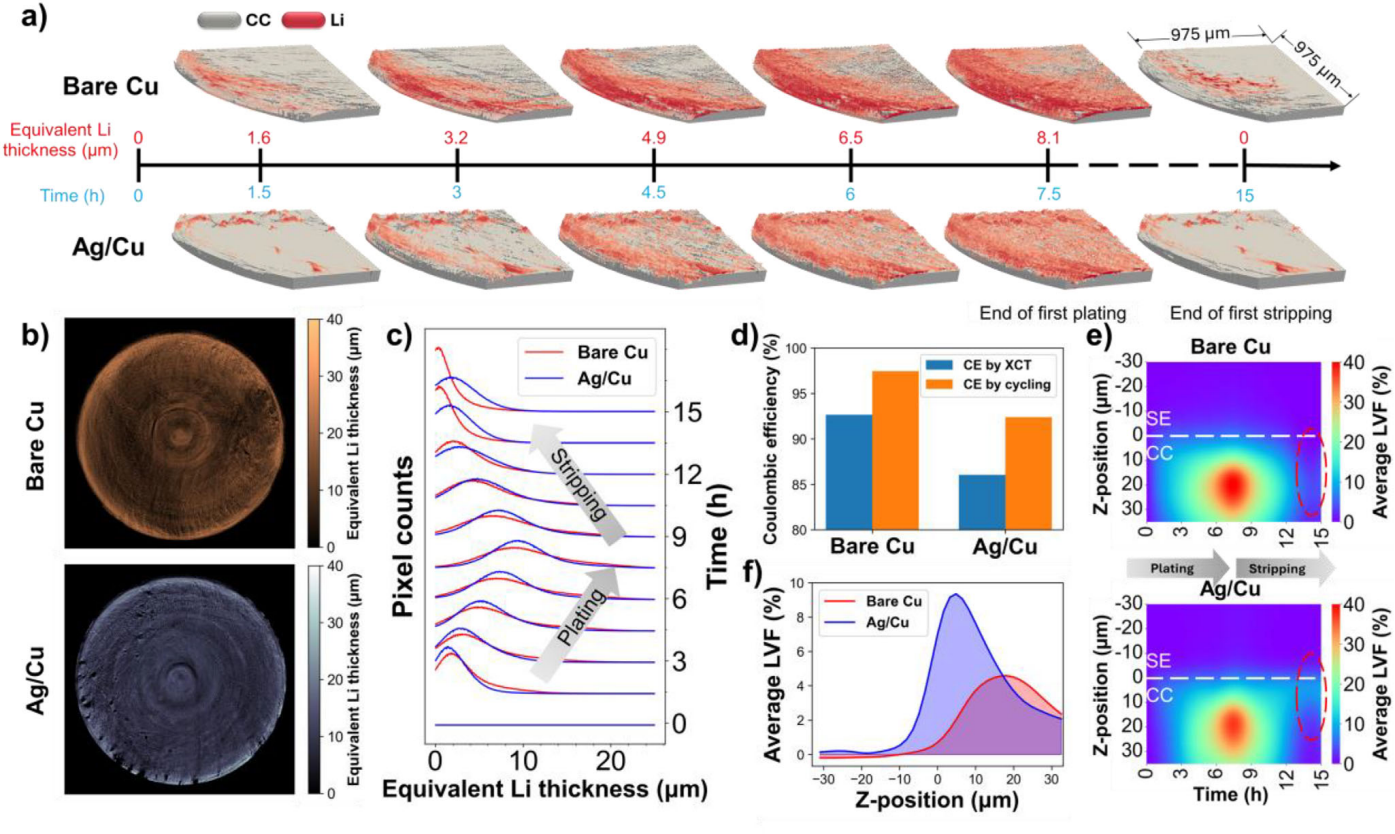
Operando XCT data analysis on first plating and stripping a low current density of 0.25 mA cm^−2^_(WE)_ performed on Li|LPSC|Cu and Li|LPSC|Ag/Cu cells. (a) The 3D volume rendered images showing the morphology evolution of plated lithium on bare Cu and Ag/Cu. (b) In‐plane distribution of the equivalent lithium thickness on Cu and Ag/Cu current collectors at the end of the first plating. (c) Thickness frequency distribution of equivalent lithium thickness on the bare Cu and Ag/Cu current collectors during the first plating/stripping. (d) Coulombic efficiency comparison calculated by XCT and cycling data. (e) Average LVF evolution at different through‐plane positions near the SE|Cu and SE|Ag/Cu interfaces during the first plating and stripping. (f) Average LVF at different through‐plane positions near the SE|Cu and SE|Ag/Cu interfaces after the first plating‐stripping cycle.

The LVF image enables precise quantification of the plated and stripped lithium on the CC within the entire ASSB stack. Summing the LVF values along the through‐plane (Z) direction within a selected region of interest (ROI) spanning 40 pixels (≈65 µm) near the SE|CC interface produces equivalent lithium thickness maps (e.g., Figure 5b), and average thickness values. Figure 4c compares the lithium thickness evolution in the Li|LPSC|Cu and the Li|LPSC|Ag/Cu cells calculated from the LVF images and that extracted from the electrochemical cycling data. The strong agreement between the independent measurements confirms the robustness and advantage of ISM‐based image processing for quantitative analysis on plated and stripped lithium in our zero‐excess ASSB configuration.

### 2.4 Lithium Visualization and Quantification at Low Current Density

During the first plating and stripping cycle at a low current density of 0.25 mA cm^−2^_(WE)_, neither the Li|LPSC|Cu nor Li|LPSC|Ag/Cu cells exhibited short circuits, as previously noted (Figure 1c,d). However, the morphology evolution of the plated and the stripped lithium suggested by gray‐scale images (Figures 2 and 3) was not conclusive. Using the LVF images processing, we rendered a 3D volume visualizations of lithium distribution over the first cycle for both cells (Figure 5a), depicting the current collector in gray and the lithium in red. The lithium visibility is correlated with the LVF magnitude; higher LVF pixels appear to be opaquer, whereas lower LVF pixels are more transparent.

Distinct morphological behaviors were visualized on the two current collectors. (i) On bare copper: Lithium deposition initiates preferentially at the electrode edges by 1.5 h after plating onset (≈1.6 µm). With continued plating up to 7.5 h, lithium progressively extends toward the electrode center but remains preferentially edge‐biased and heterogeneously covers the surface of the copper. We attribute this plating behavior to uneven interface contact combined with the low wettability of lithium on the Cu_2_O‐rich surface of the copper current collector [49]. After the completion of the stripping (15 h), residual irreversible lithium persists near the edges. (ii) On Ag‐coated copper: Plating begins at preferential nucleation sites away from the edges. By 7.5 h, lithium deposition also appears at the edges, but the final lithium layer is notably more uniform than on bare copper. This difference in lithium plating morphology compared to the bare copper can be explained by the enhanced lithium wettability achieved with just 50 nm of Ag coating, which has high lithium solubility [31] and render the copper surface lithophilic. At the end of the stripping (15 h), residual irreversible lithium likewise remains on the current collector surface but is localized around the initial nucleation spots.

Although only a part of the stack was rendered and presented in Figure 5a, the same tendencies are consistent across the entire WE planes according to equivalent lithium thickness distribution maps (Figure 5b) and their temporal evolution (Figures S11 and S12). The thickness frequency distribution corresponding to the equivalent lithium thickness calculated from the bare and Ag‐coated copper current collectors reported in Figure 5c and Figure S13 further illustrate these differences, where a higher and narrower peak indicates a smaller standard deviation and thus more homogeneous lithium thickness distribution (Figure S13, Table S1). During the first plating, the cell with Ag/Cu current collector exhibits a higher peak in blue, indicating improved lithium uniformity compared to the cell with bare Cu current collector. However, during stripping, the red peak representing the bare Cu becomes sharper and taller with a lower mean value. Indeed, the position of the distribution peaks after stripping (Figure S13b) and corresponding statistical analysis (Table S1) show that the first‐cycle irreversible (inactive) lithium amount is higher on Ag/Cu, correlating with the lower Coulombic efficiency values in Li|LPSC|Ag/Cu relative to Li|LPSC|Cu as reported in the histogram of Figure 5d. This trend of the first‐cycle irreversibly stripped lithium aligns with the electrochemical cycling data despite some discrepancy in absolute Coulombic efficiency values (Figure 5d), attributed to the void formation after stripping. As a matter of fact, voids yield to low GSVs similar to lithium and contribute to the ISM‐processing results. The differences in lithium plating uniformity and stripping irreversibility can also be visualized in Figure 4c, where the shaded area indicates the range of equivalent thickness, characterized by the mean value and standard deviation of the thickness distribution.

The higher first‐cycle irreversibility upon lithium stripping from Ag/Cu, which results in lower CE compared to bare Cu, is evident not only during the operando measurements, but also more pronounced in the first cycle of the critical current density and capacity (CCCD) test (Figure S14). During the plating stage, a relatively small capacity of 0.05 mA cm^−2^(WE) was plated on both cells, sufficient only to complete the Ag‐Li alloying process. During the subsequent stripping/dealloying process on Ag/Cu, a pronounced irreversibility is observed, attributed to sluggish kinetics of the dealloying reactions, leading to a CE of only ≈40% on Ag/Cu. In contrast, the CE on bare Cu is higher (≈61%), primarily due to the formation of solid electrolyte interphase (SEI) and the presence of electronically and ionically isolated lithium. Furthermore, as discussed above (Figure S5), the alloying signatures during the initial plating process progressively disappear during subsequent cycles, confirming the irreversible nature of the dealloying process in early cycles. It is important to note that the CE reported here corresponds exclusively to the first cycle, whereas during long cycling, the CE can become significantly different for the Ag/Cu current collector, as the Ag‐Li alloy is already formed after the first cycle. In a separate experiment, we investigated the effect of Ag layer thickness (i.e., 10, 50, 100, and 200 nm) on the first cycle CE (Figure S15), which clearly indicates the tendency of decreasing first cycle CE with increasing Ag thickness.

The SEI formation also influences the coulombic efficiency. Due to its narrow electrochemical stability window, LPSC undergoes reductive decomposition upon contact with lithium metal, forming species such as Li_2_S, LiCl, and Li_3_P [50, 51, 52]. These byproducts form a mixed‐conductive and mechanically fragile SEI, which increases the electrochemical irreversibility, impedes uniform lithium transport and promotes continuous interfacial reactions during cycling. Lately, coulometric titration time analysis (CTTA) has been proposed as an effective electrochemical method to quantitatively evaluate the abovementioned side reactions occurring between lithium metal anodes and solid electrolytes [14]. A recent study from our group implemented CTTA to assess the lithium consumption during SEI formation at Li|LPSC interface [53], revealing an accumulated charge loss of 12–15 µAh cm^−2^ over the time scale of this experiment (15–20 h). When compared to the first plating capacity of 1.875 mAh cm^−2^_(WE)_, the SEI‐related contribution (0.6–0.8% of total capacity) is considered minor relative to the overall irreversibility (≈3% for bare Cu and ≈7% for Ag/Cu), indicating that dealloying processes dominate. Moreover, since plated metallic lithium is expected to be in direct contact with the LPSC in both cells, a similar SEI formation mechanism is expected at both SE|CC interfaces, making the influence of SEI formation on the CE between the two cells negligible.

Figure 5e presents the average LVF evolution at different through‐plane positions (Z‐positions) for both cells. The LVF evolution spans over a 35–40 µm range near the SE|Cu and SE|Ag/Cu interfaces. Notably, the through‐plane distribution at the end of stripping (red dashed ovals in Figure 5e,f) reveals spatially distinct patterns: irreversible lithium concentrates essentially at the interface (Z = 0 µm) on Ag/Cu, suggesting that trapped lithium in the Ag alloy interlayer is not fully delithiated. Conversely, on bare Cu, residual lithium resides farther from the interface (≈20 µm), likely related to the edge deposition and mechanical constraints at the electrode rod edges.

### 2.5 Lithium Visualization at High Current Densities

**FIGURE 6:**
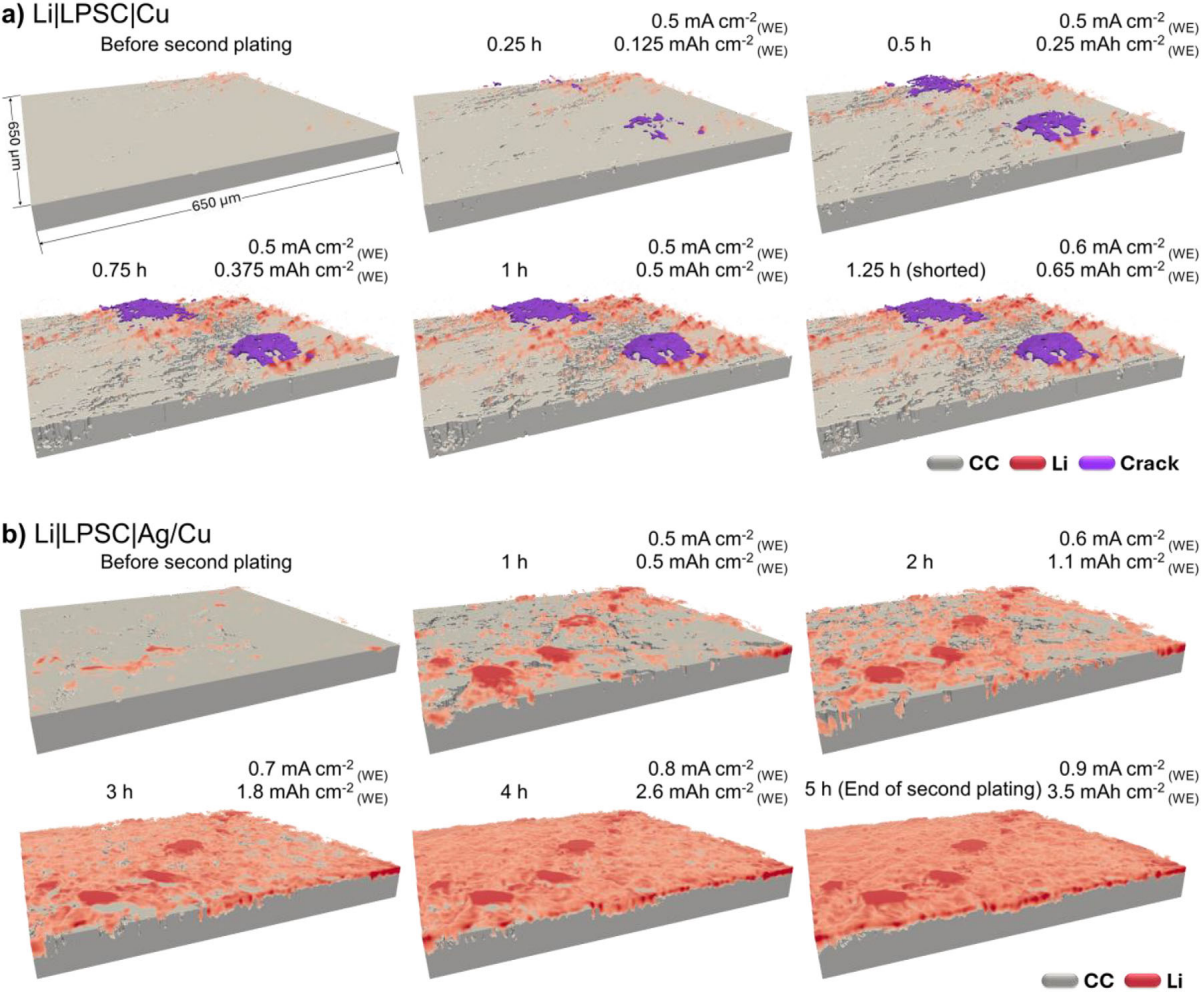
The 3D volume rendered images from the operando XCT performed at high current densities (above 0.5 mA cm^−2^_(WE)_) showing the morphology evolution of plated lithium and plating‐induced cracks on (a) on bare Cu current collector and (b) on Ag‐coated Cu current collector. Li is marked in red, the current collector is marked in gray, and cracks/infiltrated lithium are marked in purple. Associated movies are available as supporting information.

Figure 6 presents the 3D volume rendering that visualizes the lithium morphology evolution during the second plating on the WE at elevated current densities above 0.5 mA cm^−2^_(WE)_. In the rendered images, the current collector is shown in gray, plated lithium on the current collector surface in red, and the cracks with infiltrated lithium in purple.

1. On bare Cu (Figure 6a): Residual lithium from the first cycle persists on the Cu surface and acts as nucleation centers for subsequent lithium plating. In addition to those nucleation spots, new lithium deposits form isolated “islands” and grow as plating progresses. The spallation cracks initiate at 0.5 mA cm^−2^_(WE)_ after 0.25 h of plating, corresponding to an areal capacity of 0.125 mAh cm^−2^_(WE)_ or an estimated lithium thickness of ≈0.54 µm_(WE)_. The cracks initiated within the LPSC separator, slightly offset from the SE|CC interface (Figure 2b), suggesting submicron lithium dendrites, unresolved by XCT, penetrated the SE and accumulated in internal pores. Between 0.25–1 h, as plating continues reaching an equivalent lithium thickness of ≈2.16 µm_(WE)_, the cracks propagate outward from the central region toward the interface, forming basin‐shaped spallation. At a higher current density of 0.6 mA cm^−2^_(WE)_, the cell shorted after 15 min of additional plating (equivalent to 0.65 µm_(WE)_ more lithium). Despite this failure, only minimal morphological changes are observed near the crack sites, indicating the presence of nanoscale transverse cracks propagating toward the CE.
2. On Ag‐coated Cu (Figure 6b): As with the bare copper case, second‐cycle lithium plating begins around remaining lithium spots, forming also preferential islands, which likely serve as nucleation points due to their better interfacial contact and lower resistance. However, in contrast to bare copper, these lithium islands are large and grow between 50–250 µm in size after 1 h, then they merge and interconnect by 2 h (1.1 mAh cm^−2^_(WE)_), eventually forming a continuous lithium network covering the SE|CC interface by 3 h (1.8 mAh cm^−2^_(WE)_). By 4 h (2.6 mAh cm^−2^_(WE)_), the remaining gaps are filled, and by the end of the plating (3.5 mAh cm^−2(^_WE)_), a fairly homogeneous lithium layer is achieved. Throughout the high‐current‐density plating process, no spallation cracks or any other forms of failure mechanisms were captured at the SE|CC interface. The maximum applied current density reached 0.9 mA cm^−2^_(WE)_ with a total areal capacity of 3.5 mAh cm^−2^_(WE)_. This performance exceeds the CCCD tests for the Li|LPSC|Ag/Cu cell (Figure 1b), which we attribute to the improved interfacial contact between the SE and the WE during the first plating/stripping at lower current density. This highlights the importance of formation cycles [54] in mitigating void formation and enhancing long‐term interface stability.

Additionally, the thin Ag interlayer plays a critical role. Owing to its high solubility in lithium [31] it promotes more uniform lithium plating, although early‐stage deposition still occurs at preferential nucleation sites. Unlike bare Cu, however, these nucleation sites on Ag are larger from the outset and do not grow significantly in height during subsequent plating. This is attributed to the improved wettability of lithium on the Ag‐coated current collector (CC), which enables a progressively homogeneous surface coverage. As a result, the Ag layer improves SE|CC contact uniformity, helping to homogenize the local current density, suppress dendrite formation, and distribute mechanical strain more evenly. By reducing vertical strain penetration into the solid electrolyte, the Ag layer also helps prevent crack initiation. Two movies available as supporting information, recorded for both bare copper and Ag‐coated copper, enable real time tracking of lithium morphology evolution and crack formation during the high current density plating.

## 3 Li|SE Interfacial Stress Accumulation

**FIGURE 7:**
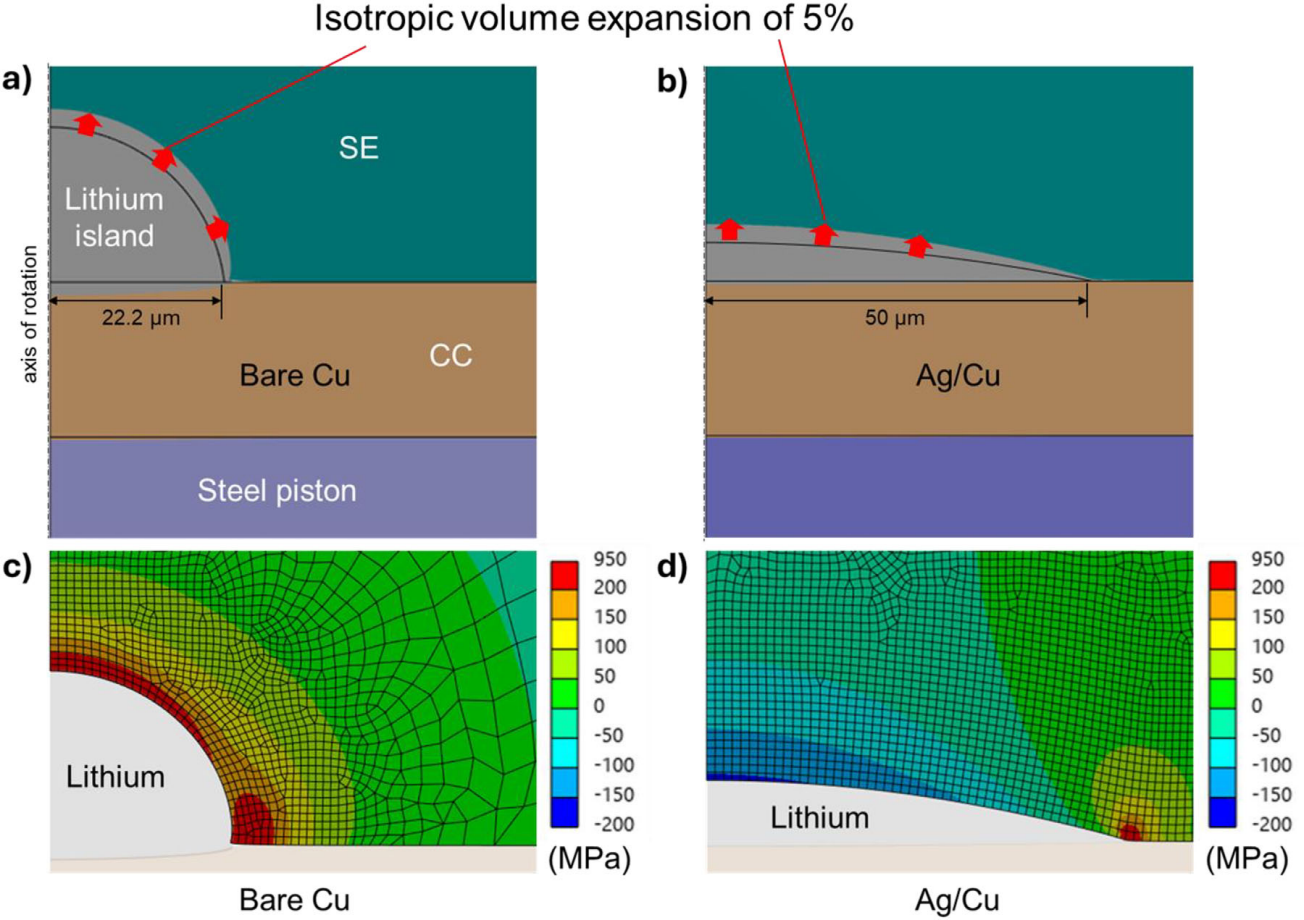
Simulation results shown with a deformation scale factor of 5 for better visibility. Deformed components in the vicinity of (a) the higher lithium island on bare copper, and (b) the flat and wide lithium island on Ag/Cu, with the black edges corresponding to the undeformed geometries. Maximum principal stress distribution in the SE around the lithium islands (c) on bare Cu and (d) on Ag/Cu.

Mechanical finite element simulations were performed to better understand the interfacial mechanical stress distribution and the resulting risk of SE cracking in the vicinity of the lithium islands. Two predefined lithium island geometries with identical total lithium volumes were compared. These two representative island shapes mimic the growth of small and large lithium deposits typically observed at the SE|Cu and the SE|Ag/Cu interfaces, respectively (Figure S16). (i) A high, narrow lithium island with a contact diameter of 44.4 µm at the current collector, exhibiting predominantly vertical growth and little lateral expansion, simulating the poor lithium wettability on copper (Figure 7a), and (ii) a flat, wide island with a contact diameter of 100 µm, showing greater lateral expansion and reduced growth in height, corresponding to improved lithium wettability on Ag (Figure 7b). The 2‐D model with axial symmetry assumes identical lithium volumes when fully revolved. The applied loads include a stack pressure of 20 MPa and an isotropic expansion coefficient of 5% to simulate progressive lithium growth. The resulting deformations in the vicinity of lithium islands are shown in Figure 7a,b. Plating‐induced cracks into the LPSC SE originate primarily from regions of high tensile stress, therefore, the maximum principal stress was evaluated. Stress values were used here for qualitative comparison between the two cases.

The simulations reveals that the narrow island on bare Cu generates high tensile stresses of several hundred MPa above the island, indicating a significant risk of cracking (region with maximum principal stress above 200 MPa are highlighted in red in Figure 7c). In contrast, the flat island on Ag/Cu shows tensile stresses only at the edges and negative stresses (i.e., compressive stresses) across almost the entire upper surface of the island (Figure 7d). These results demonstrate that a flatter, laterally expanded lithium morphology markedly reduces the risk of crack initiation and dendrite penetration into the SE.

## 4 Conclusion

In this study, we successfully performed operando XCT on asymmetric, sulfide‐based ASSB cells featuring a thin lithium CE and a copper current collector as the WE. To enable accurate quantitative analysis, we developed and validated an image subtraction method (ISM)‐based processing workflow, overcoming key challenges associated with lithium phase segmentation in XCT datasets. This workflow allowed real‐time monitoring of lithium morphological evolution during plating and stripping, as well as structural changes at both the Li|SE and SE|Cu interfaces which respectively mimic lithium metal and zero‐excess lithium ASSBs configurations.

Our work highlights the power of operando XCT for visualizing and quantifying dynamic morphological and structural changes in ASSBs. These insights provide a deeper understanding of the failure mechanisms in both lithium metal and zero‐excess lithium configurations. We demonstrate that a 50 nm Ag nucleation layer enhances Cu surface wettability, transforming lithium plating from non‐uniform, edge‐dominated growth on bare Cu to homogeneous deposition on Ag‐coated Cu. At high current densities, we reveal that the lithiophobic nature of bare copper promotes sub‐micron scale nucleation sites, driving lithium dendrites to penetrate the SE and accumulate within internal pores. This accumulation progressively builds strain in the SE, ultimately leading to spallation cracks. In contrast, lithium plating on lithiophilic Ag‐coated copper forms initially much larger nucleation sites between (50–250 µm) that do not grow significantly in height, as lithium rapidly diffuses across the surface. This promotes a more uniform and lower local current density, which helps to evenly distribute mechanical strain, reducing interfacial stress, and minimize the risk of crack initiation. Consequently, dendrite penetration into the solid electrolyte is effectively suppressed, as validated by mechanical finite element simulations.

More importantly, our results identify a vital design principle for next‐generation batteries, by revealing the critical influence of nucleation nanolayers on the lithium deposition morphology, reversibility, solid electrolyte mechanical integrity, and short‐circuit behavior under varying current densities. These findings emphasize the importance of interface engineering as a key strategy in mitigating electro‐chemo‐mechanical degradation in zero‐excess lithium all‐solid‐state batteries.

## 5 Experimental Section

### 5.1 Material Preparation and Cell Assembly

Li_6_PS_5_Cl (LPSC, NEI Corporation, particle size 3–5 µm), employed as solid electrolyte for all the cells, was ball‐milled for 1 h at 140 rpm using 5 ZrO_2_ balls (10 mm) with 500 mg of LPSC powder in a FRITSCH Planetary Micro Mill. Copper foil (Goodfellow, 20 µm thick) was punched into 3‐mm‐diameter discs and used as current collectors (CC). The copper was used as received or after 50 nm silver deposition by DC‐magnetron sputtering using a LEICA EM ACE200 vacuum coater.

6.0 mg of LPSC powder was pressed uniaxially at 380 MPa for 1 min inside a 3‐mm‐diameter PEEK tube using a stainless‐steel die, forming a 3‐mm‐diameter LPSC pellet as separator with a thickness of 500 µm. A 3‐mm‐diameter current collector (either bare Cu or Ag‐coated Cu) was then pressed onto one side of the LPSC pellet. A 2‐mm‐diameter of thin (50 µm) metallic lithium coated on copper foil (13 µm thick) obtained from China Energy Lithium Co., LTD was punched out and pressed onto the opposite side of the LPSC pellet. The entire cell stack was then compressed at 25 MPa for 1 min using a torque wrench to ensure good interfacial contact. During galvanostatic plating and stripping, a stack pressure of 20 MPa was applied. All assembly steps were conducted entirely inside an Ar‐filled glovebox.

### 5.2 Electrochemical Measurements

Galvanostatic cycling was conducted for the lithium plating and stripping during the operando X‐ray computed tomography (XCT) measurements, using an Ivium CompactStat2.h potentiostat. For low‐current operation, both cells were cycled with a current density of 0.25 mA cm^−2^_(WE)_ and an areal capacity of 1.875 mAh cm^−2^_(WE)_ (half‐cycle duration: 7.5 h, with lithium equivalent plating and stripping thickness of 8.1 µm). For high‐current operation, current densities were stepwise increased from 0.5 to 0.9 mA cm^−2^_(WE)_, with a plating time of 1 h for each step. During high‐current operation the Li|LPSC|Cu cell shorted at 0.6 mA cm^−2^_(WE)_ during lithium plating on the copper, while the Li|LPSC|Ag/Cu cell survived the lithium plating on the copper current collector up to 0.9 mA cm^−2^_(WE)_ and it shorted during the lithium stripping from the copper current collector at the same current density of 0.9 mA cm^−2^_(WE)_, with voltage limits set between −2.5 –2.5 V. In the constant‐current constant‐duration (CCCD) test performed in the laboratory, the current density was increased in 0.05 mA cm^−2^_(WE)_ increments starting from 0.05 mA cm^−2^_(WE)_, with half‐cycle duration of 1 h, until short circuits occurred, with voltage limits set between −0.2–0.2 V. The electrochemical measurements under laboratory conditions were conducted using a BioLogic MPG‐200 potentiostat.

### 5.3 Operando Synchrotron X‐ray Computed Tomography (XCT)

Operando XCT measurements were conducted at the I13‐2 beamline at the Diamond Light Source (Experiment No. MG33261‐1). A pink beam (mean energy: 28 keV) was generated by tuning the undulator gap to 5 mm. This setup maximized the X‐ray beam flux and improved the time resolution. Projections were collected using a pco.edge 5.5 sCMOS camera, combined with a 500 µm thick LuAG lens and a 2× intermediate tube lens, resulting in a total 4× magnification. This configuration provided a voxel size of 1.625 × 1.625 × 1.625 µm^3^ and a field of view of 4.2 × 3.5 mm^2^. The exposure time was set to 100 ms. For each tomogram, 2560 equiangularly distributed projections were taken over 180°, along with 40 dark and 40 flat field references, resulting in a total acquisition time of approximately 4.5 min. The projections were reconstructed using the Paganin phase retrieval method and a filtered back projection algorithm implemented in Savu. X‐ray tomograms were obtained in the pristine state and during galvanostatic plating and stripping. The scanning frequency was 2 scans per hour at low current density operation, and 4 scans per hour at higher current densities operation. A tomogram was collected for each cell at the end of each plating/stripping step.

### 5.4 Data Processing and Analysis

All image processing and analysis were performed using a custom Python script unless stated otherwise. Prior to subtraction, the selected images stacks from different time points were aligned using built‐in rigid registration function in Fiji ImageJ, referencing a stable region in the bulk LPSC separator near the SE|CC interface. A Gaussian filter (σ = 1.0) was applied to reduce the image noise. The difference images were then computed by subtracting the post‐cycling tomogram from the pre‐cycling tomogram.

LVF images: The difference images were divided by a constant normalization factor, calculated as the average grayscale value (GSV) difference between the current collector phase (sampled from current collector near the SE|CC interface) and the Li phase (sampled from the Li foil electrode), generating the images of lithium volume fraction (LVF). The LVF images were further denoised by a median filter with a radius of 3 voxels. The LVF images (for example, in Figure 4a‐5) describe the volume fraction of lithium within each pixel. Therefore, the range of LVF values can vary from 0 to 100%, as indicated by the colorbar, with 0 standing for completely non‐plated pixels (blue) and 100% standing for fully plated pixels (red).

Equivalent lithium thickness: The calculation of “equivalent lithium thickness” was done within a cylindrical region of interest (ROI) with a radius of 1495 µm and a height of 65 µm around the SE|CC interface (the height and position of such an ROI is indicated with orange rectangle in Figure S17), which essentially covers the SE|CC interfacial region of the entire stack. All the LVF values within the ROI were summed along the vertical axis (*Z*‐axis) to obtain the equivalent lithium thickness at different in‐plane positions and thereby the in‐plane thickness distribution. The ROI includes the plated lithium layer, part of the SE, and part of the CC, as shown in Figure S17. However, as the LVF values are calculated based on image subtraction, the SE and CC parts with essentially unchanged grayscale values (GSVs) have LVF values close to zero, and thus very little contribution to the summed LVF values. By statistical analysis of all the thickness values throughout the entire plane, histogram distributions (data range: 0–45 µm; number of bins: 500) were used to comprehensively visualize the data frequencies of different lithium thicknesses throughout the plane, for instance, in Figure S13. The lithium thickness should be non‐negative (≥ 0 µm) and hardly exceeding 25 µm (as indicated in Figure S13a) over the first cycle. Therefore, 45 µm was considered as a safe upper limit, and “0‐–45 µm” was selected as the data range to include all the effective thickness values in this experiment. In addition, the number of bins has been selected as a fairly high number, “500”, to ensure a smooth distribution tendency of the equivalent lithium thickness. The temporal evolution of such histograms was visualized in Figure 5c to illustrate the changes in the frequency distribution of equivalent lithium thickness upon cycling, in which the histogram profiles were plotted with lines (red lines stand for bare Cu and blue for Ag/Cu), whereas the shaded area under the curves was hidden for clearer visualization.

3D visualization: the current collector phase was classified by threshold‐based segmentation on the GSV. The crack phase was classified by threshold‐based segmentation on the LVF and the spatial coordinates. Within the Li phase, only voxels with LVF greater than 20% were visualized. The 3D volume rendering was produced using ParaView.

### 5.5 Scanning Electron Microscopy (SEM)

A ZEISS ULTRA 55 microscope was used to examine the surface and cross‐section morphologies of the bare Cu and Ag‐coated Cu foils. The cross‐section samples were prepared by an ion milling instrument Hitachi IM4000, using Ar‐ion beam at room temperature, with an acceleration voltage of 4 kV and milling time of 5 h for the LPSC pellet and 2 h for the metallic foils. All electrodes were transferred from the glovebox into the SEM chamber under vacuum using in‐house‐built transfer chamber. All images were acquired at an acceleration voltage of 5 kV using an in‐lens detector, with a working distance between 1 and 6 mm.

### 5.6 Mechanical Finite Element Simulation

The simulation of the 2‐D model with rotational symmetry was realized using Ansys Mechanical 2025 R1 software. It was meshed using 2D quad elements with 8 nodes and a global element size of 10 µm. A mesh refinement was implemented in the region of the lithium island (element size 1 µm) in order to resolve the higher stress gradients correctly (Figure S18). An isotropic, bilinear elastic‐plastic material model was used for components where plastic deformation was expected, and an isotropic, linear‐elastic model for the rest. All material properties were taken from the literature [55, 56, 57] (Table S2), except for the strain hardening modulus. These values were estimated based on experience but have only a minor impact on the evaluated results. The Ag layer is not physically included in the simulation.

The boundary conditions and loads are highlighted in Figure S19. These include rotational symmetry, the boundary conditions resulting from the experimental set‐up, i.e., the surrounding PEEK tube, the applied stack pressure, and the expansion of the lithium island. Frictional contacts were defined between all components to allow relative movements at the interfaces. An exception is the SE|CC contact, which was defined as bonded (Figure S20) because, even with the growth of the lithium island, no peeling off between SE|CC could be detected in the experiments.

## Conflicts of Interest

The authors declare no conflict of interest.

## Supporting information




**Supporting File 1**: smll72076‐sup‐0001‐SuppMat.docx

**Supporting File 2**: smll72076‐sup‐0001‐Movie S1.mp4

**Supporting File 3**: smll72076‐sup‐0001‐Movie S2.mp4

## Data Availability

The data that support the findings of this study are openly available in Zenodo (DOI: 10.5281/zenodo.15807466).
