# Supplementary material for: Operando X‐Ray Computed Tomography Reveals the Role of Interfacial Nucleation Nanolayers in Suppressing Mechanical Failure in Zero‐Excess Lithium All‐Solid‐State Batteries
Source: Small. 2026 Jan 8;22(10):e12284. doi: 10.1002/smll.202512284 (PMC12910424; doi:10.1002/smll.202512284)
Supplement: Supplementary file 1 — Supporting File 1: smll72076‐sup‐0001‐SuppMat.docx [file SMLL-22-e12284-s001.docx]

**Operando X-ray Computed Tomography Reveals the Role of Interfacial Nucleation Nanolayers in Suppressing Mechanical Failure in Zero-Excess Lithium All-Solid-State Batteries**

Linfeng Xu^1^, James Le Houx^2,3^, Vyacheslav Kachkanov^4^, Jinsong Zhang^1^, Robin Norbert Wullich^1^, Matthias Fankhauser^5^, Kaspar Löffel^5^, Thomas J. Schmidt^1,6^, Mario El Kazzi^1*^

Affiliation(s):

^1^ PSI Center for Energy and Environmental Sciences, Paul Scherrer Institute, 5232 Villigen, Switzerland

^2^ ISIS Neutron and Muon Source, Rutherford Appleton Laboratory, Didcot, UK

^3^ The Faraday Institution, Harwell Science and Innovation Campus, Didcot, UK

^4^ Diamond Light Source, Rutherford Appleton Laboratory, Didcot, UK

^5^ University of Applied Sciences Northwestern Switzerland (FHNW), 5210 Windisch, Switzerland

^6^ Institute for Molecular Physical Science, ETH Zurich, CH-8093 Zurich, Switzerland

Corresponding author: mario.el-kazzi@psi.ch


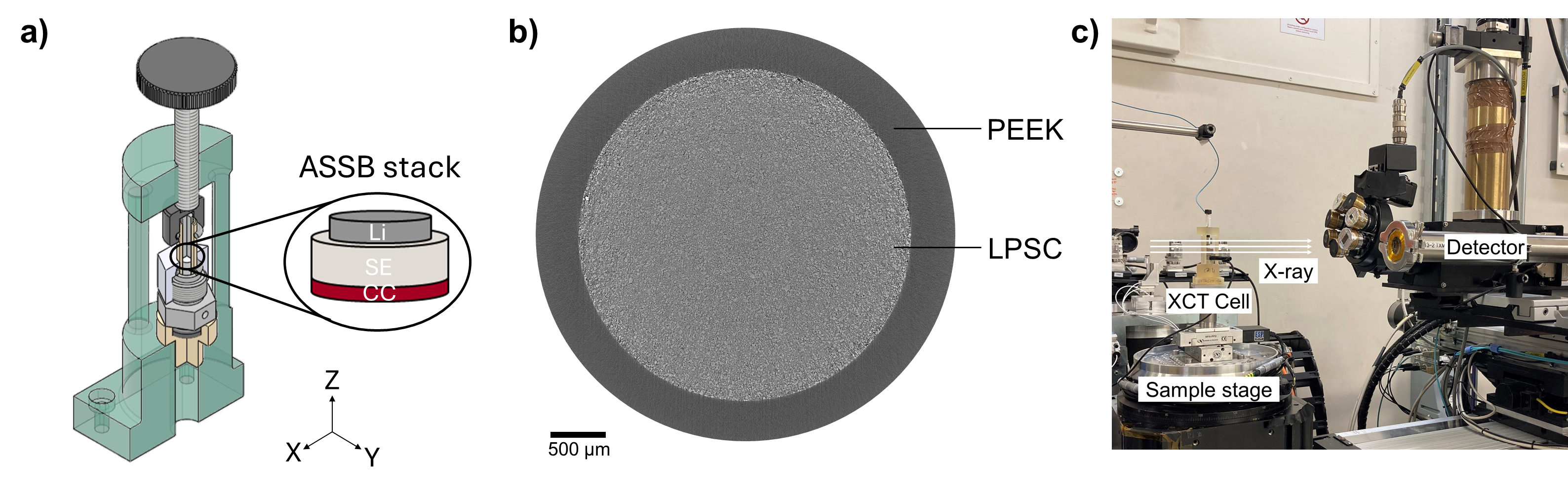


Figure S 1 (a) Scheme of the in-house designed operando tomography cell. The axis perpendicular to the electrode plane is denoted as Z-axis (through-plane), and the XY plane is parallel to the electrode plane (in-plane). (b) An original in-plane tomography slice of the Li|LPSC|Cu cell at SE position before cycling. No artifacts associated with the three PMMA pillars of the cell are observed. (c) Experimental setup of the operando XCT measurement at I13-2 beamline, Diamond Light Source.


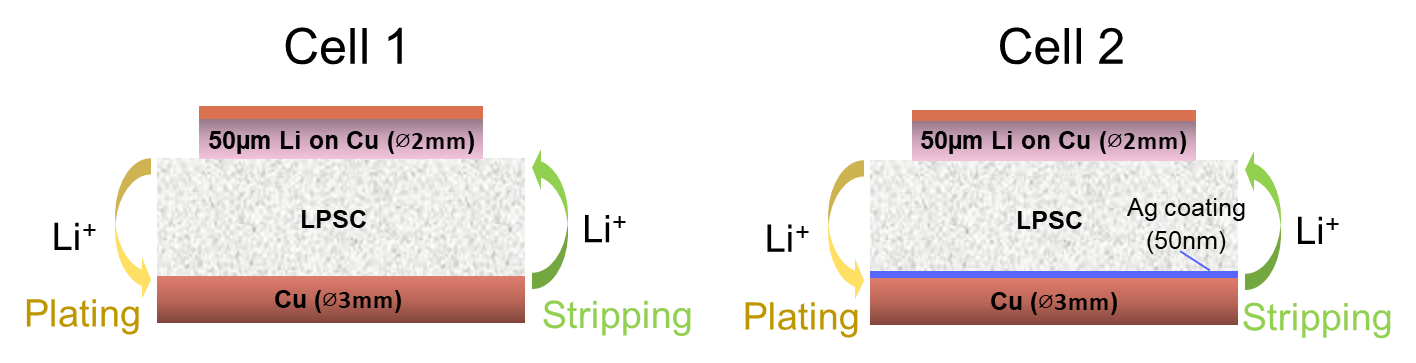


Figure S 2 The ASSB stack architectures of the two cells measured with operando XCT: Li|LPSC|Cu (left) and Li|LPSC|Ag/Cu (right). In this work, the terminology “plating” and “stripping” refer to corresponding processes on the copper current collector, unless specified.


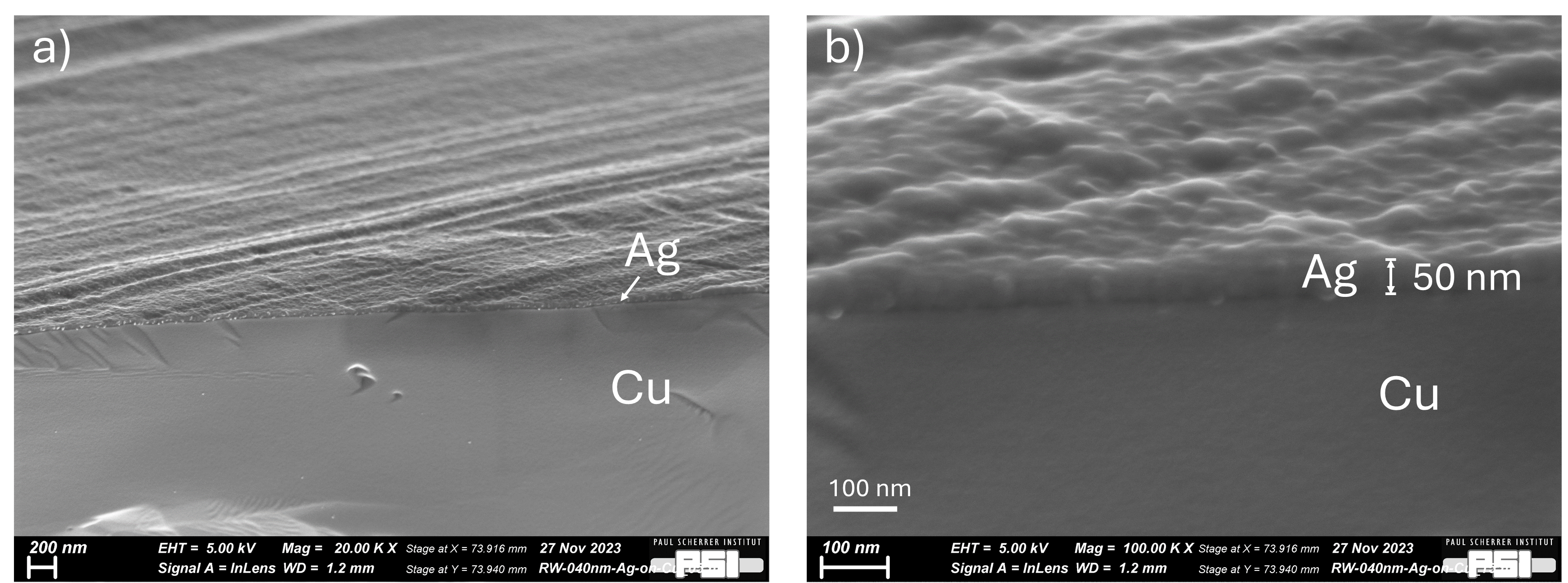


Figure S 3 Cross-section SEM images performed on the 50 nm Ag-coated Cu foil with (a) 20k× and (b) 100k× magnification.


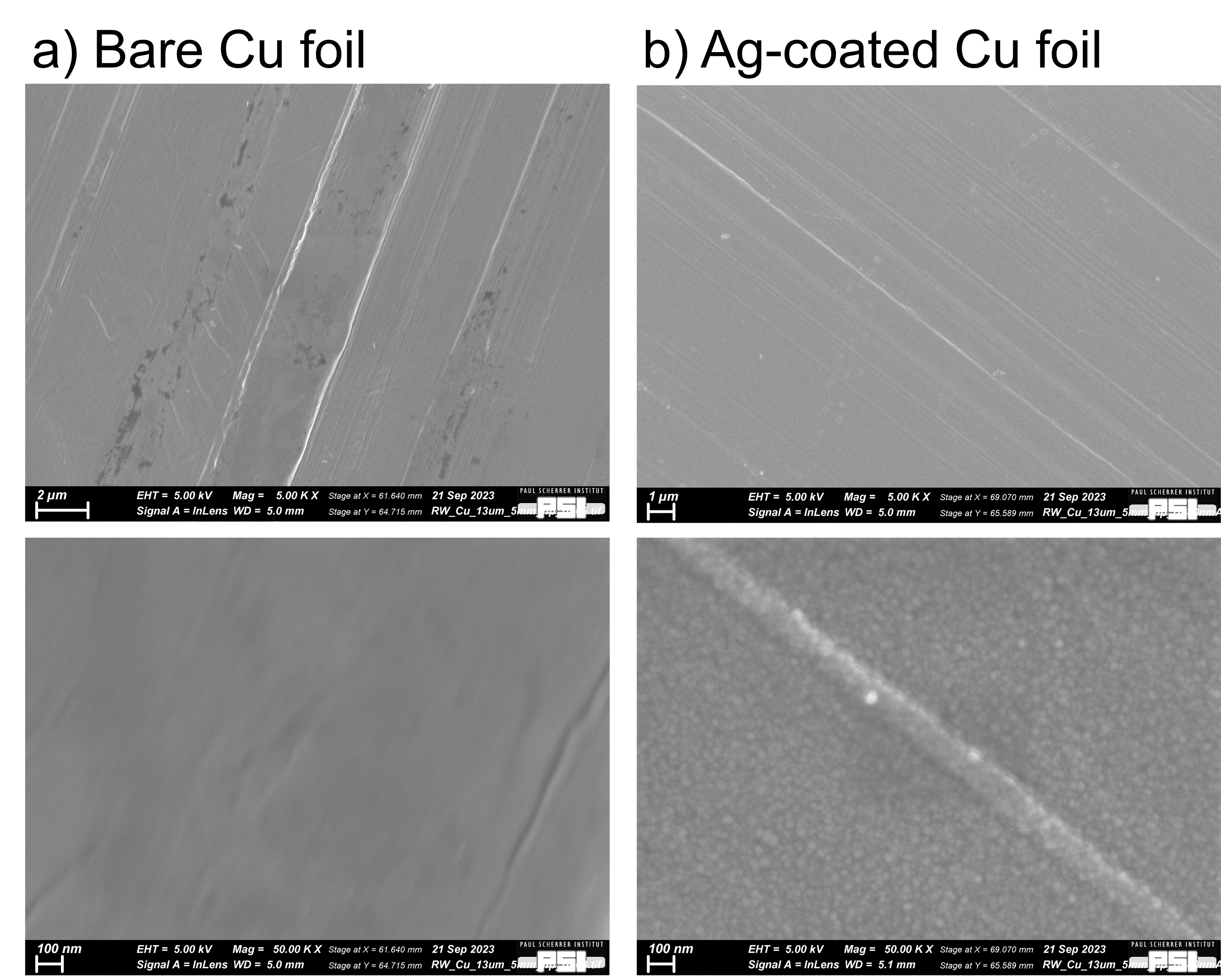


Figure S 4 Plan view SEM images performed on (a) bare and (b) 50 nm Ag-coated Cu foils with 5k× (top row) and 50k× (bottom row) magnification.


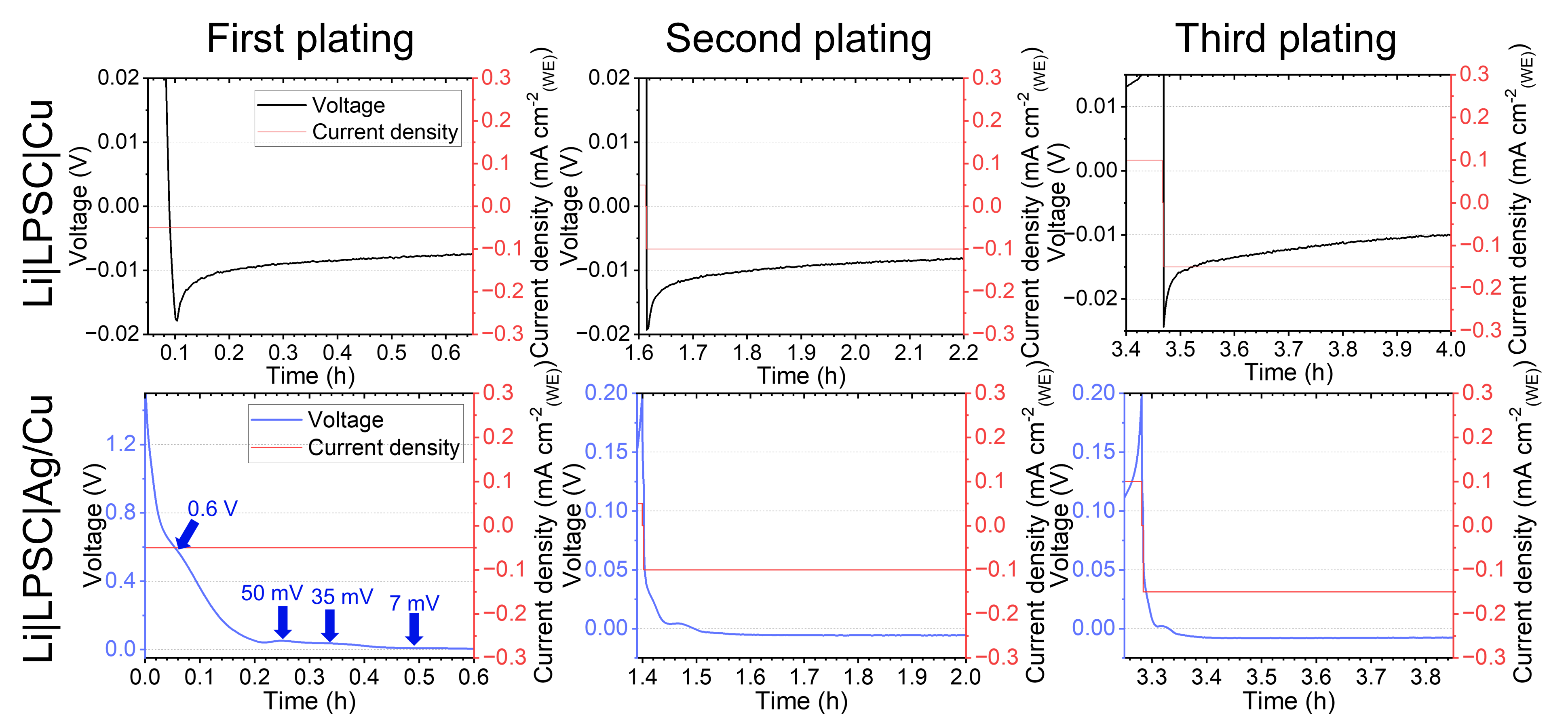


Figure S 5 Magnified voltage profiles of the first three plating during the critical capacity and current density (CCCD) test of the Li|LPSC|Cu and Li|LPSC|Ag/Cu cells, with the blue arrows indicating different voltage plateaus at approximately 0.6 V, 50 mV, 35 mV, and 7 mV assigned to Ag-Li alloy redox processes. The voltage limits are set between -0.2 V to 0.2 V.


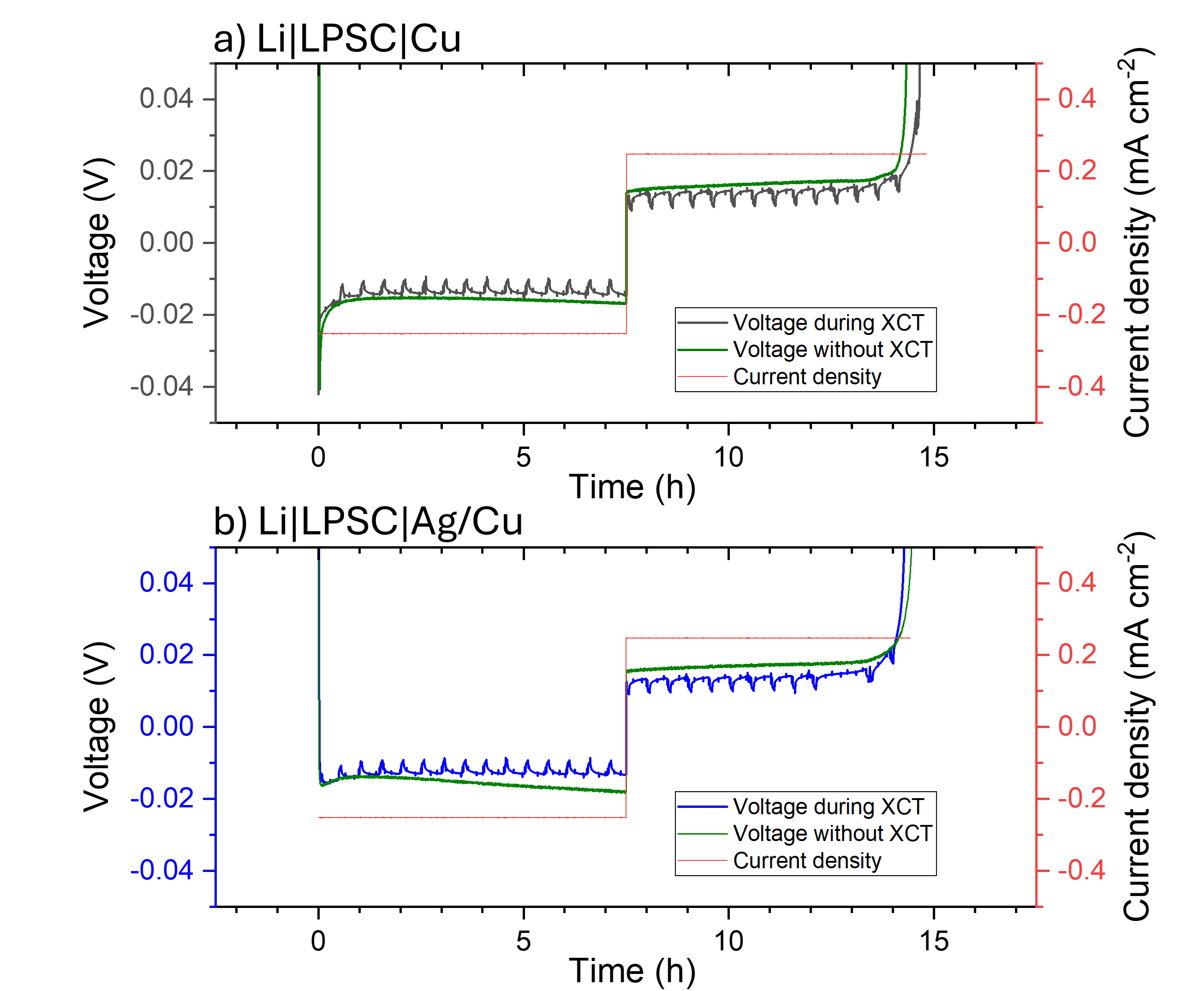


Figure S 6 Voltage profiles during galvanostatic plating and stripping performed on (a) Li|LPSC|Cu and (b) Li|LPSC|Ag/Cu cells. Comparison between the cells operating during the operando XCT and without XCT measurements shows that the periodic voltage spikes resulted by the periodic XCT scan acquisition.


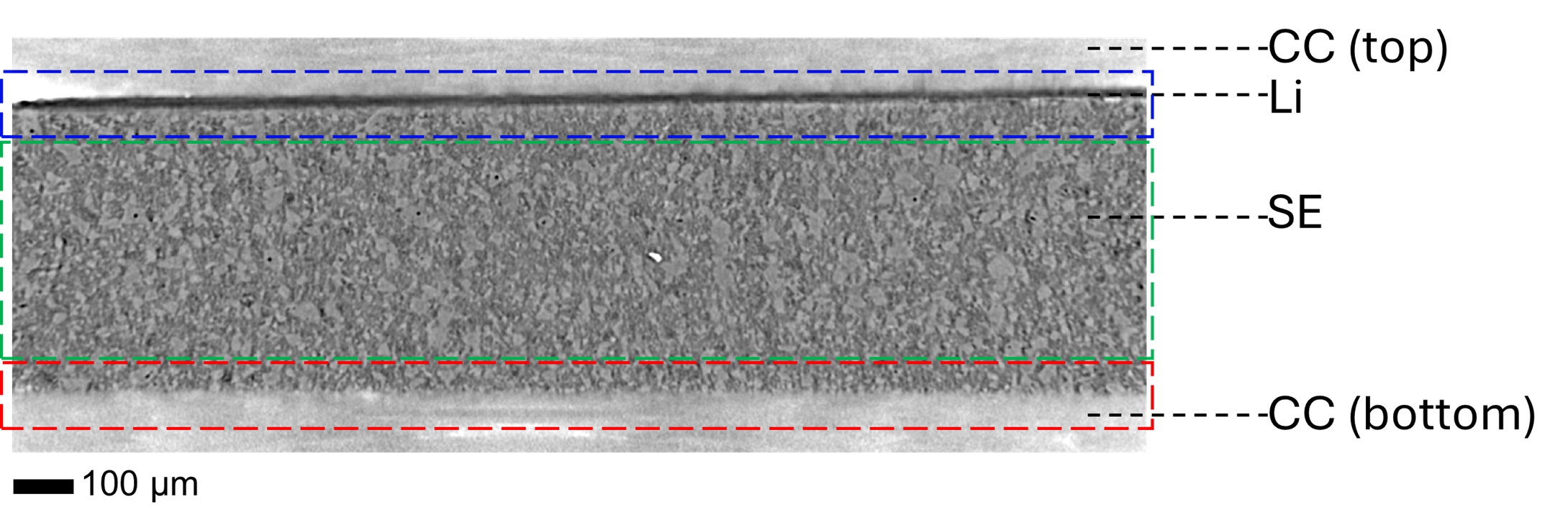
Figure S 7 A cross-sectional XCT image slice of the Li|LPSC|Cu cell, emphasizing the different phases including Li, SE, and CC (including the Cu foil and the stainless steel dies), in the stack with different GSV ranges: GSV_Li_ < GSV_SE_ < GSV_CC_ (pixels with higher GSVs appears to be brighter in the image). The blue, green, and red dashed boxes mark respectively, the interfacial region near the Li electrode, the bulky region of the LPSC separator, and the interfacial region near the copper current collector.


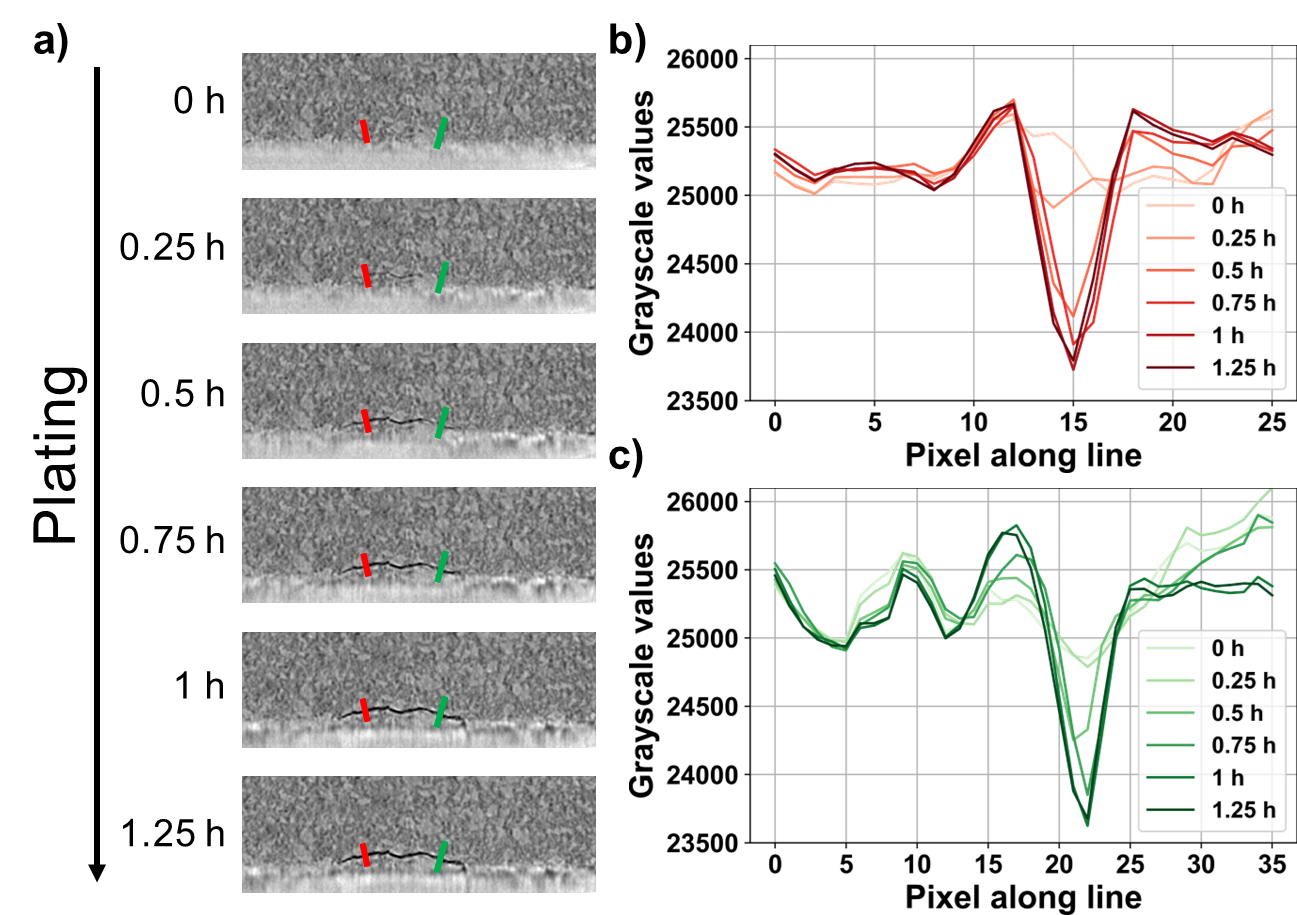


Figure S 8 The GSV line profiles taken at two different positions in (a) the XCT image slices of the Li|LPSC|Cu cell during the second plating, within a region of interest comprising one of the spallation cracks. Line profile evolution during plating is presented in (b) for the position marked in red and (c) the position marked in green, respectively.


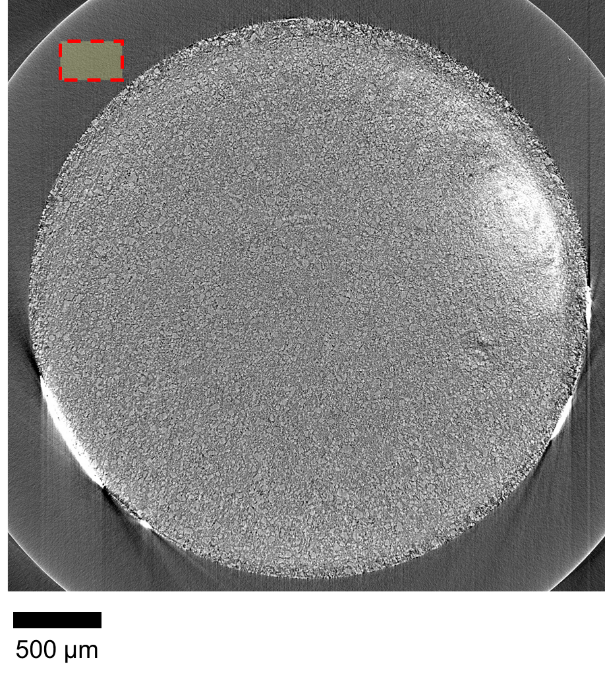


Figure S 9 An in-plane XCT slice image of the Li|LPSC|Cu cell. The red dashed box with shaded area indicates the inactive PEEK region used for testing scan stability through the entire operando measurements.


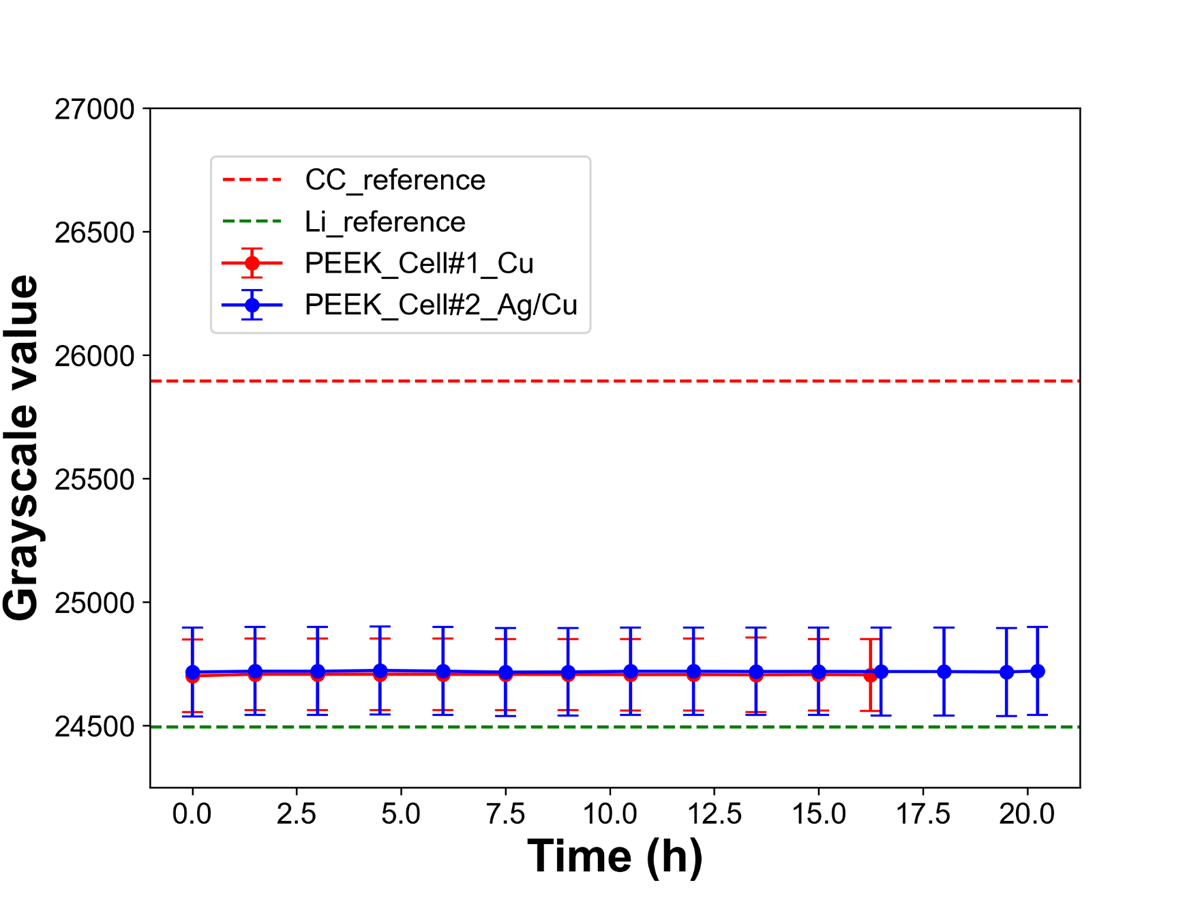


Figure S 10 Verification of the scan stability over the entire operando XCT measurements. GSVs are counted within an inactive region (selected from the PEEK tube area around the operando ASSB stacks), with error bars indicating the variation range of the GSVs (blue stands for the PEEK region in the Li|LPSC|Cu cell and red for the Li|LPSC|Ag/Cu cell). The red and green dash lines represent the average GSV of the CC and Li phases, respectively.


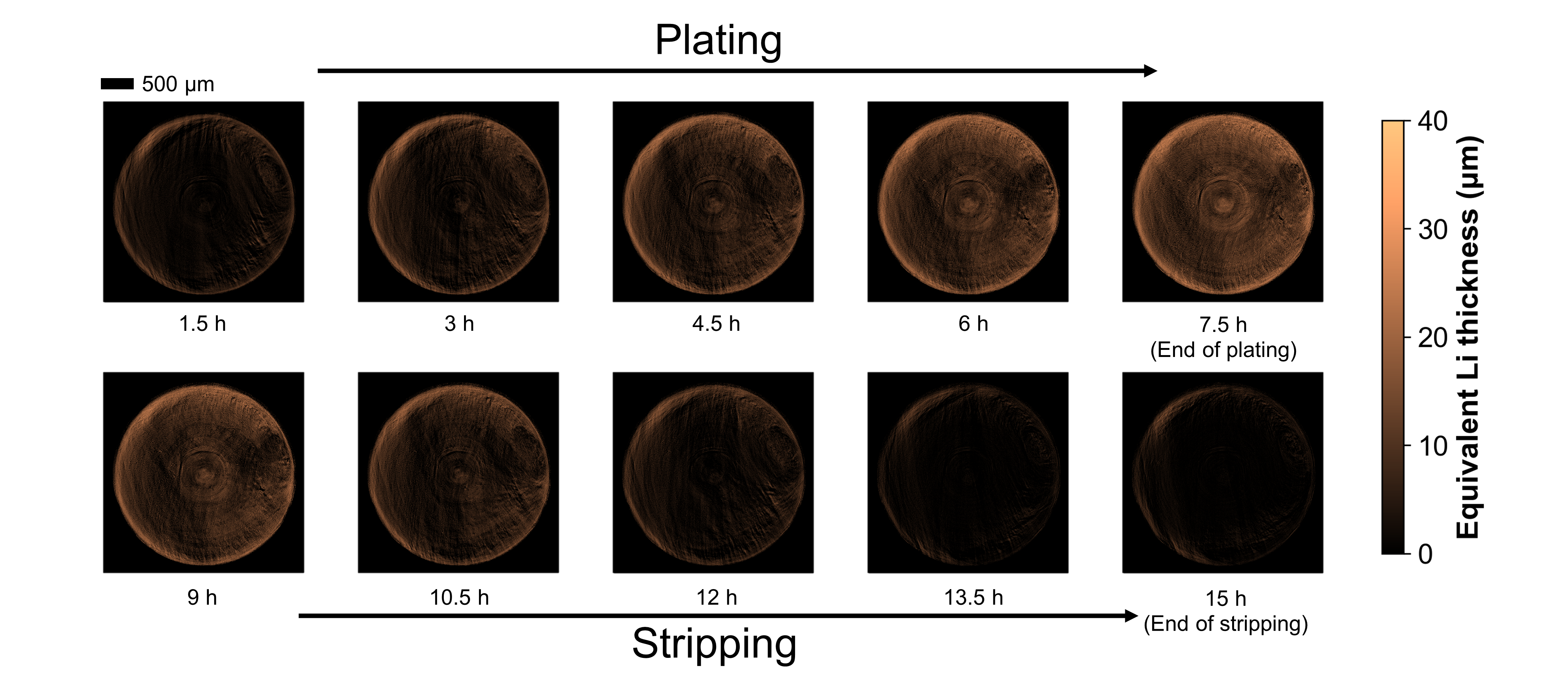


Figure S 11 Evolution of the in-plane distribution of the equivalent Li thickness on bare Cu current collector during the first plating and the first stripping at low current density of 0.25 mA cm^-2^_(WE)_.


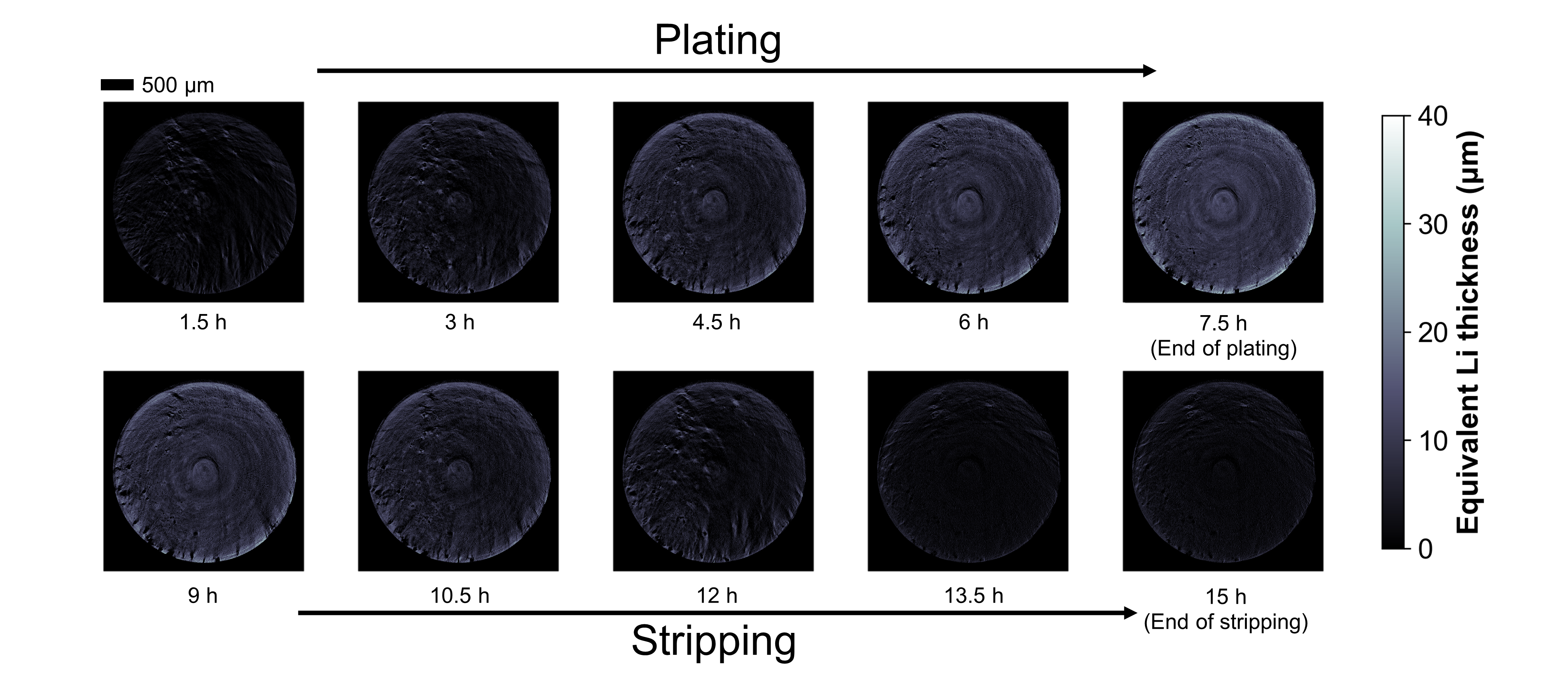


Figure S 12 Evolution of in-plane distribution of the equivalent Li thickness on Ag-coated Cu current collectors during the first plating and the first stripping at low current density of 0.25 mA cm^-2^_(WE)_.


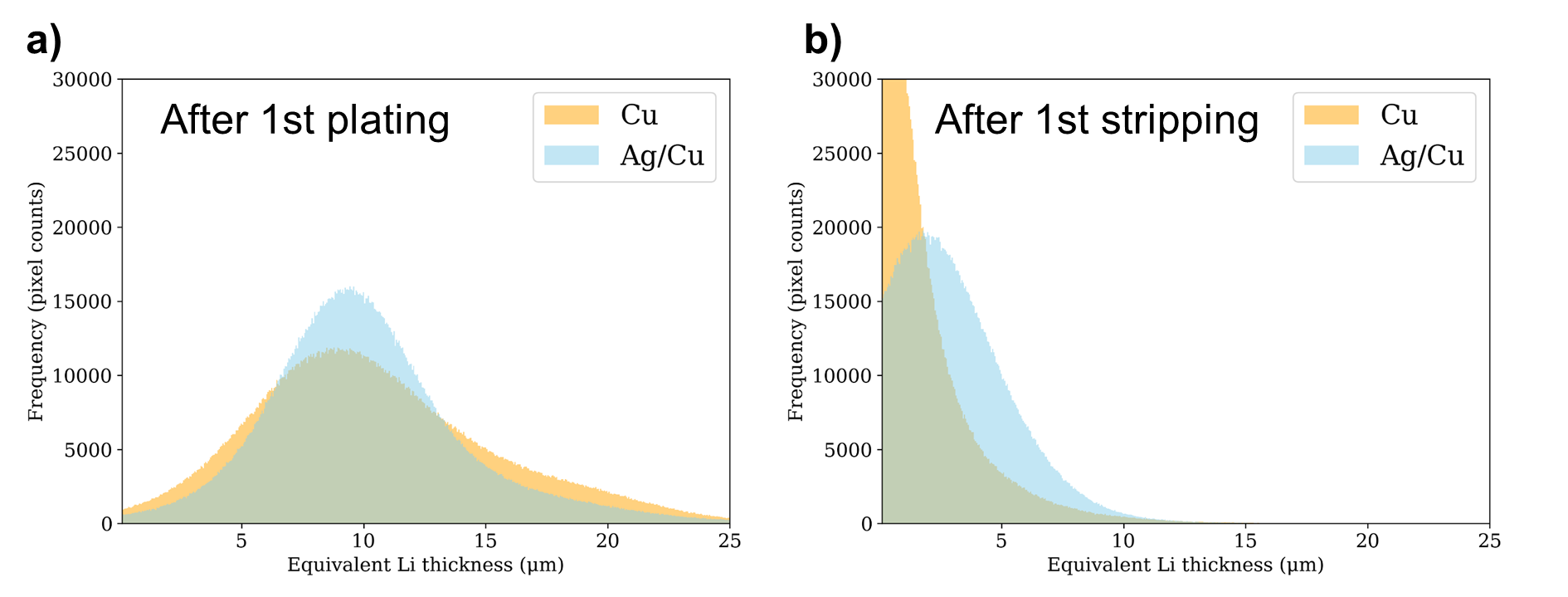


Figure S 13 Thickness frequency distribution of the equivalent Li thickness in both Li|LPSC|Cu and Li|LPSC|Ag/Cu cells after the (a) first plating and (b) first stripping, calculated from the corresponding LVF images.

Table S 1 The mean values (μ) and standard deviations (σ) of the lithium thickness distribution on bare Cu and Ag-coated Cu current collectors after the first plating and first stripping.

| Cell | *μ_after_plating_* (µm) | *σ_after_plating_* (µm) | *μ_after_strippng_* (µm) | *σ_after_stripping_* (µm) |
| --- | --- | --- | --- | --- |
| Li\|LPSC\|Cu | 9.40 | 5.31 | 0.70 | 2.29 |
| Li\|LPSC\|Ag/Cu | 8.82 | 5.03 | 1.45 | 3.09 |


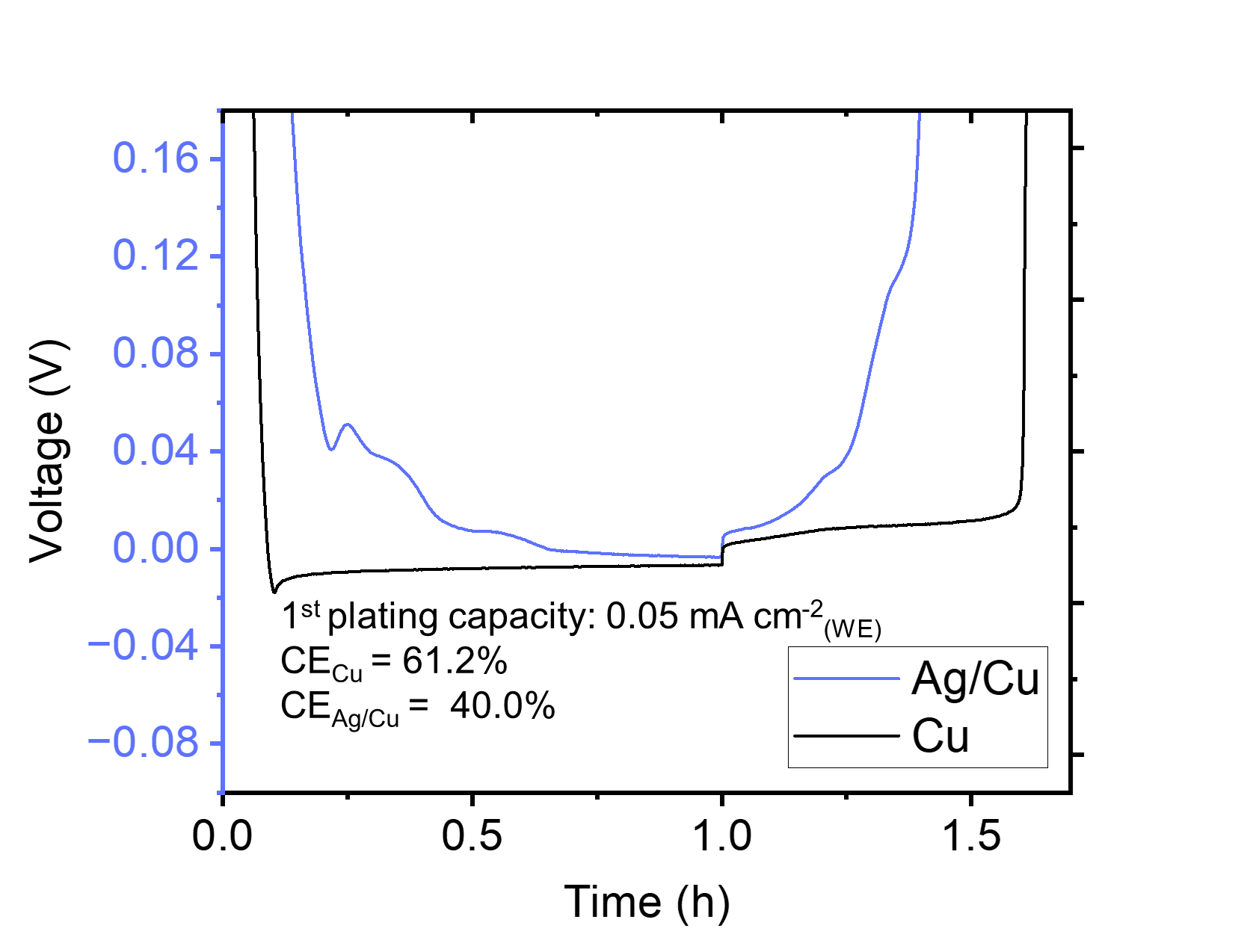


*Figure S 14 Magnified voltage profiles of the first cycle during the critical capacity and current density (CCCD) test of the Li|LPSC|Cu and Li|LPSC|Ag/Cu cells. The voltage limits are set between -0.2 V to 0.2 V.*


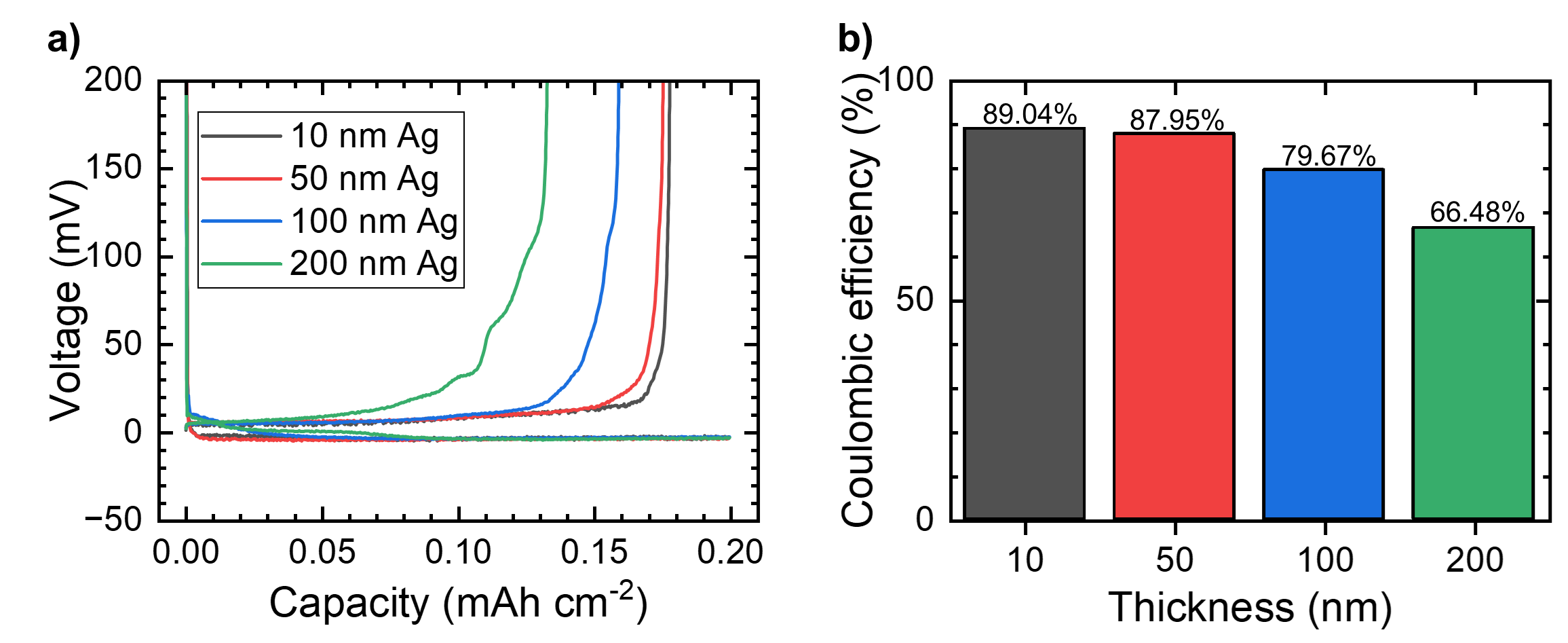


*Figure S 15 The effect of Ag layer thickness on the first cycle Coulombic efficiency. (a) Voltage profiles during the first cycle of Li|LPSC|Ag/Cu cells with Ag layer thicknesses of 10 nm, 50 nm, 100 nm, and 200 nm, where a current density of 0.05 mA cm^-2^ and an areal capacity of 0.2 mAh cm^-2^ were applied. (b) First cycle coulombic efficiency of the four cells in (a).*


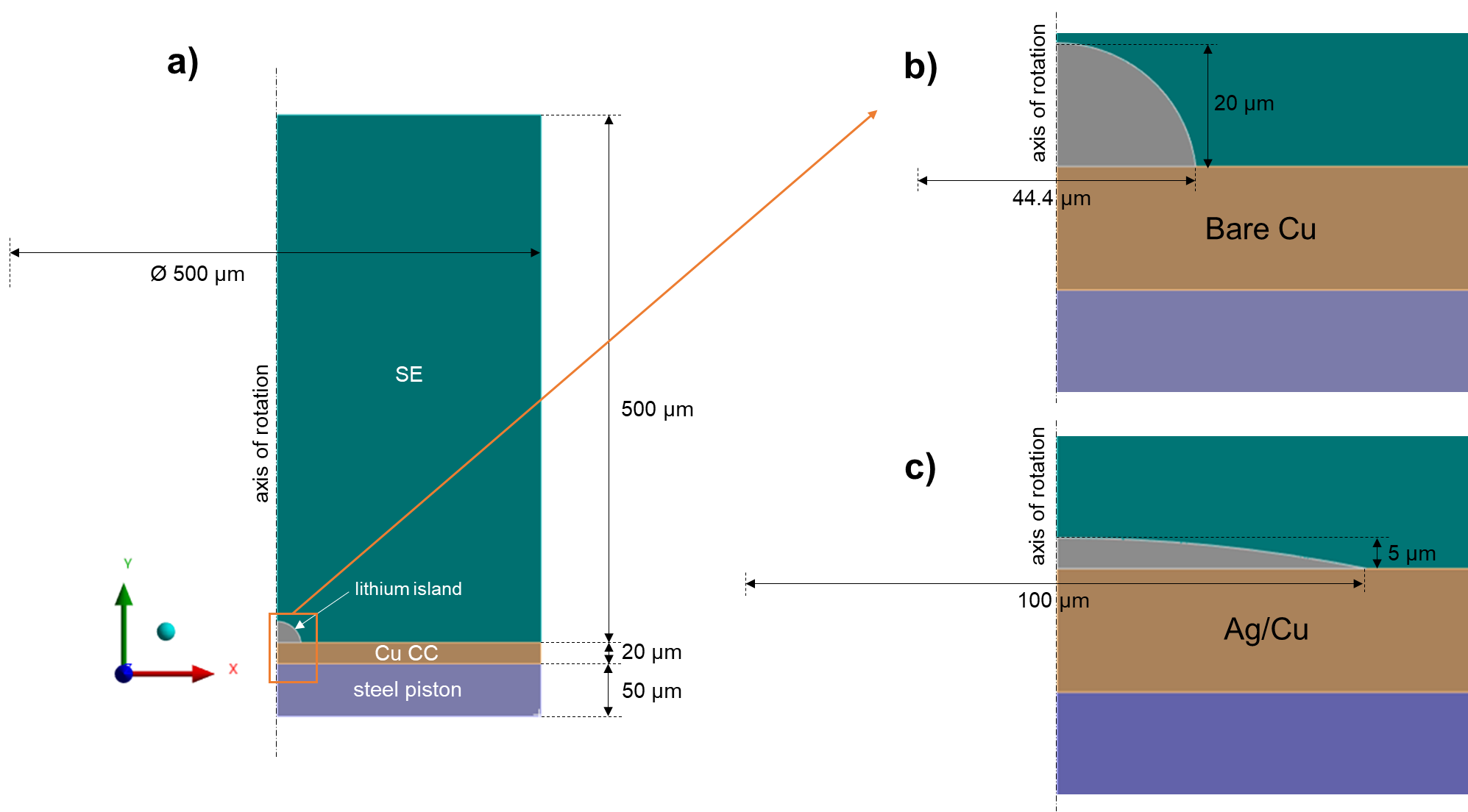


Figure S 16 (a) Dimensions of 2-dimensional simulation model with axial symmetry. (b) Size and shape of the lithium island on bare Cu which has grown predominantly in height. (c) Size and shape of the lithium island on Ag/Cu which has not grown significantly in height but laterally and has the same volume as that of the island on bare Cu. The Ag layer is not physically included in the simulation.


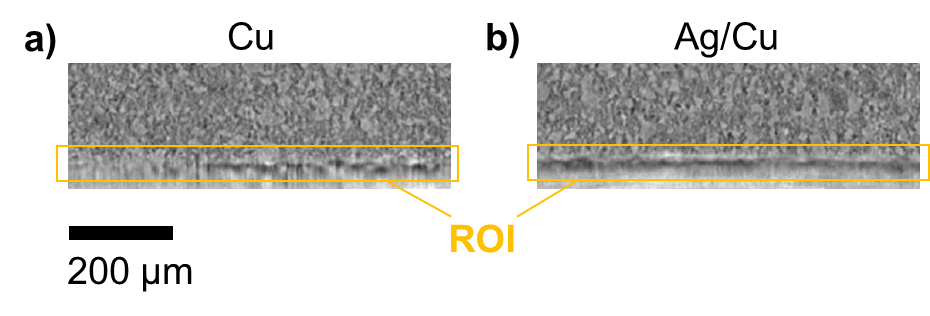


*Figure S 17 Cross-sectional tomography slices in the vicinity of the (a) SE|Cu and (b) SE|Ag/Cu interfaces after the first plating, with the orange rectangles indicating the ROI selected for LVF-related quantitative analysis in this work.*


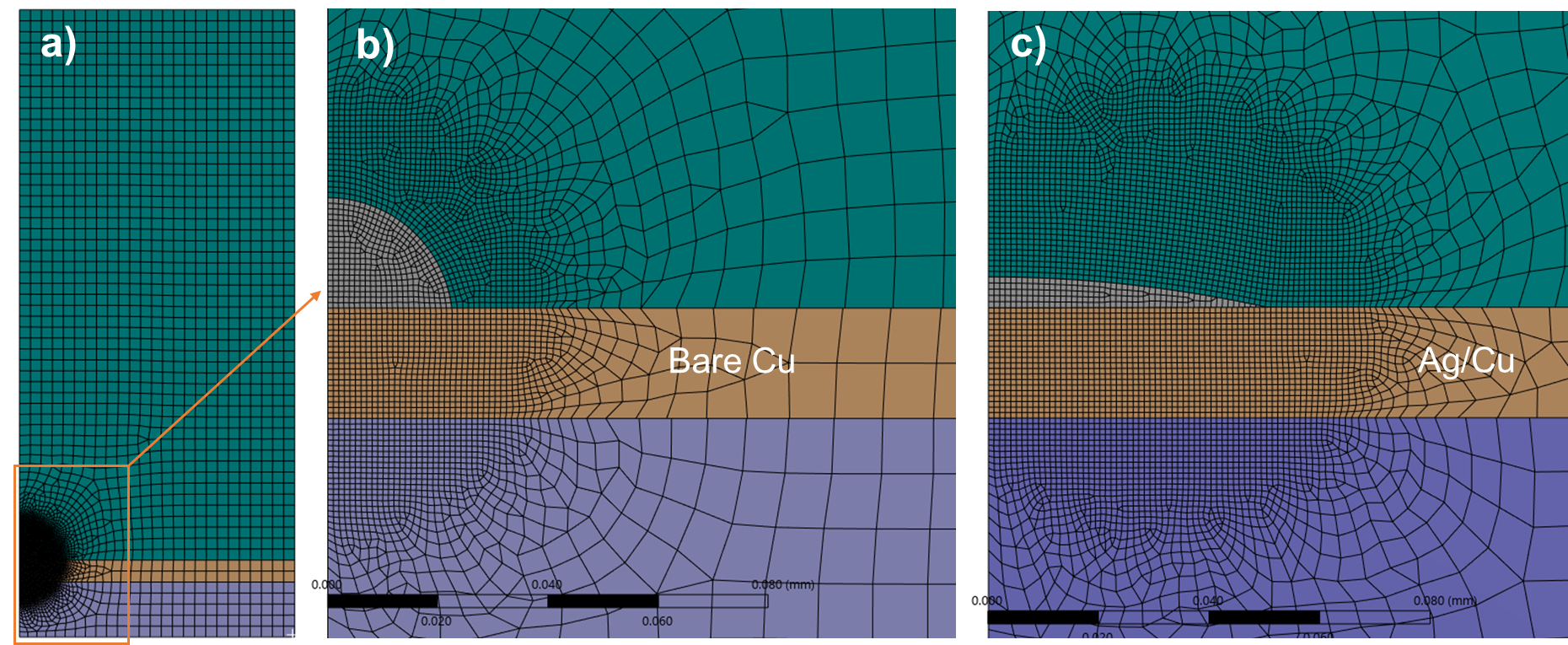


Figure S 18 (a) Finite element mesh of the complete 2D model with predominantly QUAD8 elements. (b) Mesh refinement in the region of the lithium island on bare Cu. (c) Mesh refinement in the region of the lithium island on Ag/Cu.

Table S 2 Material data used in mechanical finite element simulation

| **Material** | **Material model** | **Properties** | **Comment** |
| --- | --- | --- | --- |
| LPSC | isotropic, linear-elastic | Poisson’s ratio: 0.35  Young’s modulus: 26 GPa | brittle material and expected to fail without significant plastic deformation |
| Lithium metal | isotropic, bilinear elastic-plastic | Poisson’s ratio: 0.381  Young’s modulus: 7.82 GPa  Yield strength: 1 MPa  Strain hardening modulus: 1 GPa  Isotropic Secant Coefficient of Expansion: 0.05 | relatively low yield strength and large deformation expected due to expansion; therefore elastic-plastic model used |
| Copper | isotropic, bilinear elastic-plastic | Poisson’s ratio: 0.35  Young’s modulus: 120 GPa  Yield strength: 270 MPa  Strain hardening modulus: 20 GPa | plastic deformation expected; therefore elastic-plastic model used |
| Steel | isotropic, linear-elastic | Poisson’s ratio: 0.29  Young’s modulus: 210 GPa |  |


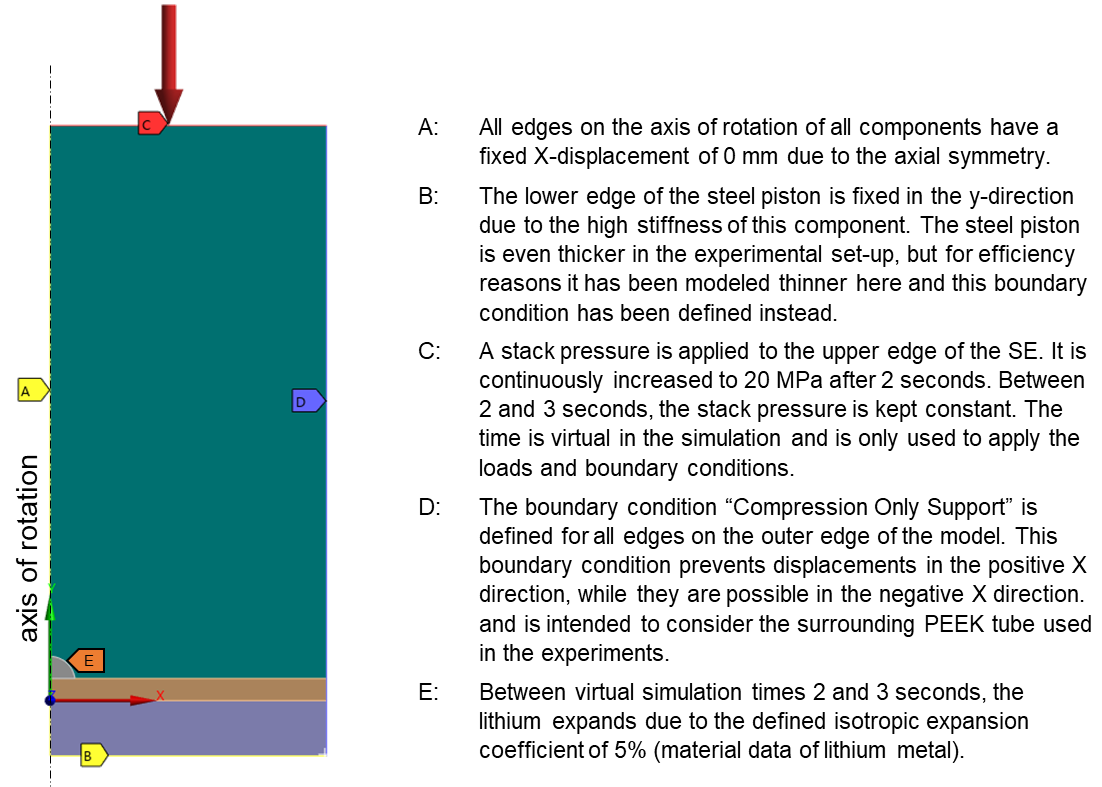


Figure S 19 Boundary conditions and loads defined in the finite element simulation


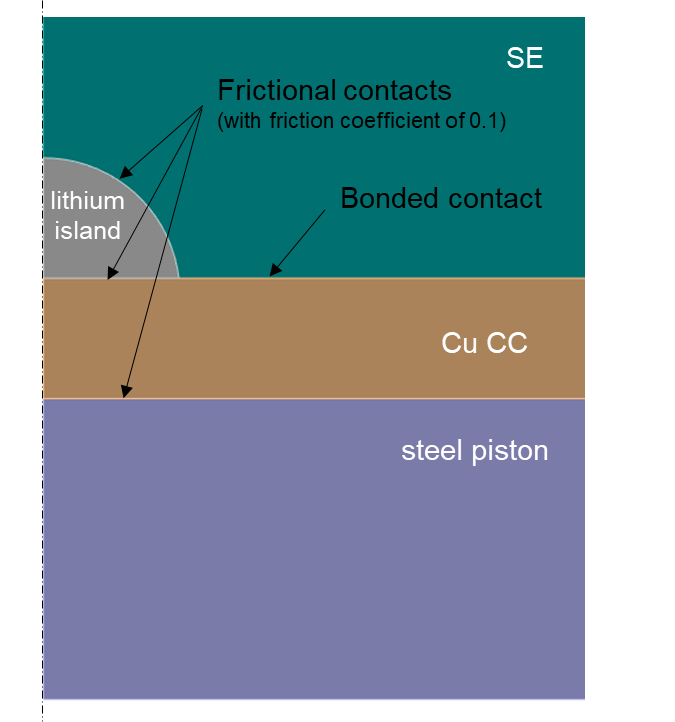


Figure S 20 Contact definition in the finite element simulation
